# Mechanisms of resistance to targeted therapy and immunotherapy in non-small cell lung cancer: promising strategies to overcoming challenges

**DOI:** 10.3389/fimmu.2024.1366260

**Published:** 2024-04-09

**Authors:** Yuchu Xiang, Xudong Liu, Yifan Wang, Dawei Zheng, Qiuxing Meng, Lingling Jiang, Sha Yang, Sijia Zhang, Xin Zhang, Yan Liu, Bo Wang

**Affiliations:** ^1^ West China Hospital of Sichuan University, Sichuan University, Chengdu, China; ^2^ Institute of Medical Microbiology and Hygiene, Faculty of Medicine, University of Freiburg, Freiburg, Germany; ^3^ College of Life Sciences, University of Chinese Academy of Sciences, Beijing, China; ^4^ State Key Laboratory for Oncogenes and Related Genes, Division of Cardiology, Renji Hospital, School of Medicine, Shanghai Jiao Tong University, Shanghai Cancer Institute, Shanghai, China; ^5^ The College of Life Science, Sichuan University, Chengdu, China; ^6^ Department of Laboratory Medicine, Liuzhou People’s Hospital, Liuzhou, China; ^7^ Guangxi Health Commission Key Laboratory of Clinical Biotechnology (Liuzhou People’s Hospital), Liuzhou, China; ^8^ Guangxi Medical University Cancer Hospital, Nanning, China; ^9^ Institute of Pharmaceutical Science, China Pharmaceutical University, Nanjing, China; ^10^ Cancer Center, Union Hospital, Tongji Medical College, Huazhong University of Science and Technology, Wuhan, China; ^11^ Zhongshan Hospital of Fudan University, Xiamen, Fujian, China; ^12^ Department of Organ Transplantation, Guizhou Provincial People’s Hospital, Guiyang, Guizhou, China; ^13^ Department of Urology, Guizhou Provincial People’s Hospital, Guiyang, Guizhou, China

**Keywords:** non-small cell lung cancer, targeted therapy, immunotherapy, mechanisms of drug resistance, promising strategies

## Abstract

Resistance to targeted therapy and immunotherapy in non-small cell lung cancer (NSCLC) is a significant challenge in the treatment of this disease. The mechanisms of resistance are multifactorial and include molecular target alterations and activation of alternative pathways, tumor heterogeneity and tumor microenvironment change, immune evasion, and immunosuppression. Promising strategies for overcoming resistance include the development of combination therapies, understanding the resistance mechanisms to better use novel drug targets, the identification of biomarkers, the modulation of the tumor microenvironment and so on. Ongoing research into the mechanisms of resistance and the development of new therapeutic approaches hold great promise for improving outcomes for patients with NSCLC. Here, we summarize diverse mechanisms driving resistance to targeted therapy and immunotherapy in NSCLC and the latest potential and promising strategies to overcome the resistance to help patients who suffer from NSCLC.

## 1 Introduction

Lung cancer is a common cancer and one of the leading causes of cancer deaths (1–4). It can be classified into two main groups based on pathologic features: small cell lung cancer (SCLC) and non-small cell lung cancer (NSCLC). NSCLC accounts for 80-85% of all lung cancer cases (5). It can be further divided into three main subtypes: adenocarcinoma (40%), squamous cell carcinoma (25-30%), and large cell carcinoma (5-10%) (6). According to the World Health Organization (WHO), the 5-year survival rate of NSCLC is only 5-10% (7).

Common therapies for NSCLC include surgical intervention, radiation, chemotherapy, targeted therapy, and immunotherapy. Surgical resection is the most trustworthy and efficient approach for managing patients with NSCLC in terms of diagnosis, staging, treatment, and palliative care. For this method to be feasible, the cancer must be fully resectable (8). In addition, it is worth noting that around 70% of individuals diagnosed with NSCLC have either locally progressed or metastatic disease. Chemotherapy induces remission in people with locally advanced and metastatic illnesses (9). While chemotherapy is suitable for numerous NSCLC patients, the utilization of traditional chemotherapeutic drugs has reached a therapeutic plateau. Radiotherapy is a conventional therapeutic method that includes stereotactic RT and hadron therapy as the primary approaches. Hadrons are subatomic particles composed of quarks, such as protons, neutrons, or heavy ions, which are influenced by powerful nuclear forces.

Although conventional treatments have been the standard of treatment, the increasing clinical use of targeted therapies and immunotherapy has provided new therapeutic ideas for a wide range of healthcare professionals. However, the emergence of drug resistance during treatment remains a thorny issue (10).

Targeted therapy is drugs that specifically target genetic alterations or signaling pathways involved in tumor growth and survival. These therapies have revolutionized the treatment of lung cancer, particularly NSCLC (11). Nevertheless, tumor cells can develop resistance to these medications via several mechanisms, including targeted modifications and activation of alternate pathways. The former refers to the ability of cancer cells to generate novel genetic modifications or mutations that provide resistance to specific therapies, such as epidermal growth factor receptor (EGFR), anaplastic lymphoma kinase (ALK), or proto-oncogene tyrosine protein kinase-1 (ROS1) mutations. The latter one refers to the observation that tumor cells can circumvent certain pathways, like mesenchymal to epithelial transition factor (MET) amplification, kirsten rat sarcoma viral oncogene (KRAS) mutations (12), that are targeted for treatment by activating alternative signaling pathways that promote the survival and proliferation of the cells. This phenomenon is commonly referred to as “adaptive resistance.” NSCLC, similar to other forms of cancer, is distinguished by its genetic and molecular diversity. Lung tumors might comprise diverse groups of cancer cells with varying genetic modifications. Although targeted medicines can effectively combat the main clone, they may not have the capability to eradicate secondary subclones that possess drug-resistant mutations (13).

Immunotherapeutic resistance refers to the ability of cancer cells to evade or overcome the effects of immunotherapy, which is a treatment approach that harnesses the body’s immune system to fight cancer (14). While immunotherapy has shown significant promise in treating NSCLC, some patients may experience resistance to these therapies (15). In the context of NSCLC, immunotherapeutic resistance can occur through several mechanisms (16–20): 1. Loss or downregulation of immune cell recognition; 2. Tumor microenvironment changes; 3. Immune checkpoint activation; 4. Tumor heterogeneity.

In this review, we review the mechanisms of resistance to targeted therapy and immunotherapy in NSCLC, and list the promising strategies for overcoming the resistance from different perspectives.

## 2 Targeted therapy resistance in NSCLC

**Figure 1:**
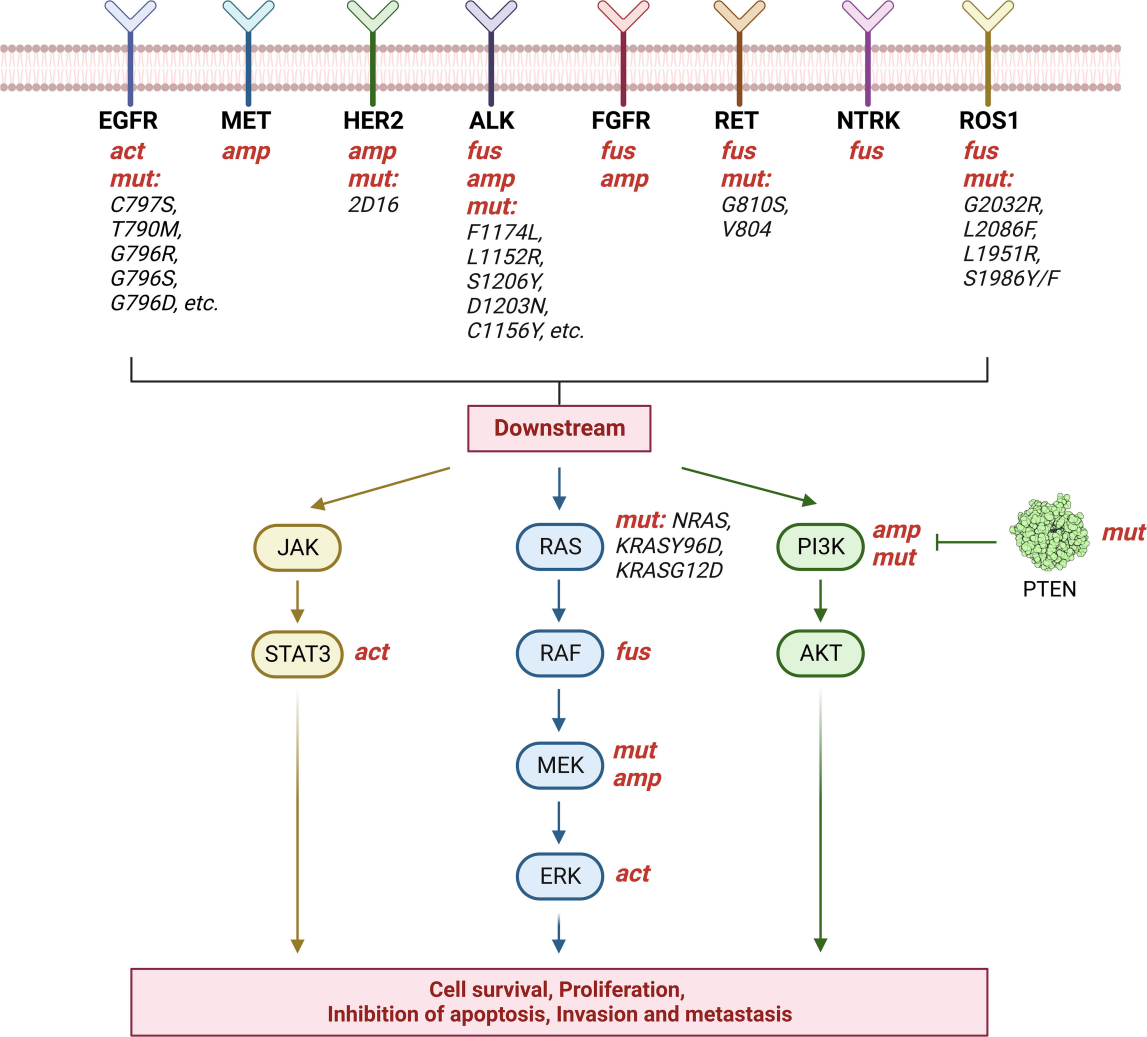
Targeted therapy resistance in NSCLC. The image shows the mechanisms of the main targets that generate resistance in targeted therapy for NSCLC. The above eight targets generate drug resistance through gene mutation(mut), activation(act), amplification(amp), or fusion(fus). Mutated targets have been marked in black. In particular, mutations in PTEN, an inhibitor of PI3K, also lead to tumor progression. Created with BioRender.com.

Herein, we summarized the mechanisms of the main targets that generate resistance in targeted therapy for NSCLC (**Figure 1**). In the clinical work, the targets can be divided into two groups: common clinical gene targets and uncommon clinical gene targets.

### 2.1 Common clinical gene targets

#### 2.1.1 EGFR: the mechanisms of resistance

The EGFR gene is the most prevalent oncogenic driver in NSCLC, occurring in 10–15% of all NSCLC cases (21). TKIs targeting EGFR mutations have emerged as the preferred first-line treatment option for patients with EGFR-mutant NSCLC. Currently, three generations of EGFR-TKI drugs have been developed. The first generation comprises gefitinib, erlotinib, and icotinib, among which the acquisition of the T790M resistance mutation is a common occurrence (22). The second generation includes afatinib and dacomitinib; however, these drugs encounter significant challenges in overcoming resistance, thereby limiting their efficacy in patients with acquired resistance to first-generation TKIs. The third generation encompasses osimertinib, rociletinib, and avitinib, and it targets resistance mutations such as EGFR L718Q, L844V, and C797S mutations.

We categorize the mechanisms of resistance generation into target-dependent and target-independent. Taking EGFR as an example, the subsequent targets will not be repeated, EGFR-dependent refers to those mediated by acquired resistance mutations in the structural domain of the EGFR kinase, whereas EGFR-independent refers to those mediated by alterations in non-target kinases, such as bypass signaling activation or phenotypic transformation (23).

1) EGFR target-dependent resistance mechanisms:

Patients receiving first- or second-generation EGFR-TKIs primarily develop EGFR target-dependent resistance, whereas only approximately 20% of patients receiving the third-generation TKI osimertinib as second-line therapy exhibit target-dependent resistance mechanisms (24).

The most common resistance mutations include the C797S mutation in exon 20 of EGFR (25) and the T790M mutation (22), which often co-occur. In cases where only the C797S mutation is present without T790M, NSCLC with drug resistance may still maintain sensitivity to quinazoline-based EGFR inhibitors such as gefitinib, erlotinib, and afatinib (26, 27). In trans-C797S/T790M cases, cells are resistant to third-generation EGFR-TKIs but remain sensitive to combined treatment with first- and third-generation TKIs (28). In cis-C797S/T790M cases, EGFR-TKI treatment alone or in combination is ineffective (25), indicating resistance to all EGFR-TKIs (29).

Various EGFR mutations have been identified as mechanisms of resistance to third-generation EGFR-TKIs (24, 30–32). These include EGFR G796R, G796S, and G796D mutations (33, 34), S768I mutations (35), L718X and L792X mutations (31), L798I mutation (36), and L858R/T790M/L792H and L858R/T790M/G796R mutations (37), which have been associated with resistance to osimertinib. Among these, the L792X mutation interferes with the spatial binding of the EGFR kinase structural domain and can coexist with trans-G796/C97X mutations (31, 37). Other rare EGFR-TKI resistance mutations include residues L718 and G719 in the Adenosine triphosphate (ATP) binding site (30, 31), as well as the G724S mutation in the P-loop structural domain of the kinase (38–40). It has been suggested that G724S is an allele-specific drug resistance mutation that occurs in the context of Ex19Del (41) and limits the activity of third-generation EGFR-TKIs both *in vitro* and *in vivo* by inducing conformational changes in the glycine-rich loop (38), but further investigation is needed (39). In addition, amplification of EGFR wild-type alleles, rather than mutant alleles, has been shown to be sufficient to generate acquired resistance (36, 42).

2) EGFR target-independent resistance mechanisms:

For EGFR target-independent resistance, MET amplification has been identified as the most common mechanism of resistance observed during treatment with osimertinib (30), followed by human epidermal growth factor receptor 2 (HER2) amplification (43).

MET-mediated resistance primarily occurs through MET gene amplification (24, 30, 36), which leads to the activation of downstream signaling pathways, including STAT, mitogen-activated protein kinase (MAPK), and phosphatidylinositol 3-kinase (PI3K), thereby bypassing the EGFR pathway. HER2, an ErbB2 receptor tyrosine kinase (RTK), also contributes to EGFR-TKI resistance by activating the MAPK and PI3K pathways. HER2 amplification has been detected in 12% of tumor samples from patients without coexisting T790M mutations treated with first-generation EGFR-TKIs (44). Moreover, HER2 exon 16 skipping deletion (HER2D16) mediates resistance to osimertinib through an Src-independent mechanism. Combining osimertinib with the pan-HER small molecule inhibitor afatinib has shown synergistic potential in overcoming HER2D16 resistance (45). Additionally, fusion events involving various oncogenes, including fibroblast growth factor receptor (FGFR) (24), B-Raf proto-oncogene (BRAF) (46, 47), ROS1 (48), ret proto-oncogene (RET) (24, 46, 47), neurotrophic tropomyosin kinase receptors (NTRK) (47), and ALK (30), have been implicated in EGFR-TKI resistance.

In addition to the resistance mechanisms mentioned previously, several other factors contribute to EGFR-induced NSCLC drug resistance. Firstly, RAS mutations, encompassing NRAS, KRAS, and BRAFV600E mutations, have been prominently associated with resistance to EGFR-TKIs (24, 30, 46). Furthermore, phosphatidylinositol 4,5-bisphosphate 3-kinase catalytic subunit alpha (PIK3CA) amplification or mutation is detected in approximately 3-5% of first-generation EGFR TKI resistance cases (30, 49) and 5-12% of third-generation TKI resistance cases (24, 30, 36). Additionally, FGFR amplification, including Src family kinase (SFK) and focal adhesion kinase (FAK), has been identified as a notable mechanism of resistance (50, 51).

Furthermore, alterations in cell cycle-related genes, such as amplification or mutation of CDK4 and CDK6, as well as CDKN2A, play a significant role in resistance development (24, 30). Moreover, histological phenotypic transformations, such as the transition to small cell lung carcinoma (SCLC), epithelial-to-mesenchymal transition (EMT), and squamous cell carcinoma transition (SCCT), also contribute significantly to resistance development. Lastly, epigenetic modifications constitute another dimension of this intricate resistance landscape.

These resistance mechanisms highlight the complexity of EGFR-TKI resistance in NSCLC, necessitating further research to improve treatment strategies and develop novel therapeutic approaches.

#### 2.1.2 EGFR: the strategies to overcome resistance

To overcome resistance to third-generation EGFR-TKIs, various strategies have been explored, including the use of first- or second-generation TKIs for EGFR target-dependent resistance. For the classical C797S/T790M mutation, clinical evidence supporting the efficacy of combination therapy with first- and third-generation EGFR TKIs has been reported (29), such as nazatinib in combination with gefitinib and osimertinib in combination with gefitinib. Studies have shown that the combination of osimertinib and gefitinib has consistent efficacy as a first-line treatment for EGFR-mutated NSCLC, which provides insights into the use of dual EGFR-TKIs (52). First- and second-generation TKIs are not affected by the C797S resistance mutation, making them potential treatment options for patients with this mutation (25). Additionally, the second-generation TKI afatinib retains its binding ability to the ATP pocket even in the presence of the G724S mutation, making it a candidate for combination therapy with osimertinib to overcome acquired G724S resistance mutations (40). Afatinib has also shown efficacy against L718Q resistance mutations (53).

Targeting non-dependent resistance to third-generation EGFR-TKIs is more challenging and often requires combination therapies.

1) EGFR-TKIs + Chemotherapy.

Combining EGFR-TKIs with chemotherapy is a common approach to delay resistance development. In the NEJ005 phase II trial, the combination of gefitinib with carboplatin plus pemetrexed demonstrated prolonged progression-free survival (PFS) and overall survival (54, 55). The NEJ009 phase III trial compared chemotherapy plus gefitinib to gefitinib alone, and the combination group showed significantly longer PFS (56). Similar conclusions were reached in other phase III trials (57). The ongoing FLAURA 2 phase III trial is evaluating the combination of carboplatin (or cisplatin) pemetrexed with osimertinib, aiming to investigate whether the observed benefits of combination therapy apply to osimertinib treatment (58).

2) EGFR-TKIs + Specific small molecule inhibitors

The combination of specific inhibitors with EGFR-TKIs, selected based on the underlying mechanism of secondary resistance, has demonstrated improved therapeutic outcomes. For instance, pralsetinib (BLU667) for RET rearrangements, brigatinib for ALK fusions, trametinib and selumetinib for MEK inhibition, entrectinib for NTRK mutations, sotorasib as a KRAS inhibitor, cabozantinib as a multi-target inhibitor, and inhibitors targeting AXL, JAK1, and BRAFV600E are potential treatment options.

MET amplification is a well-studied mechanism of drug resistance, and treatment options for MET-amplified patients have been investigated. Studies have shown that the combination of octreotide and MET-TKI-savolitinib exhibits enhanced antitumor activity, suggesting its potential as a treatment option for MET-driven EGFR TKI-resistant patients (59). Combination therapy with third-generation EGFR-TKIs and MET inhibitors is emerging as a common and promising approach for first-line treatment of NSCLC patients with MET-amplified EGFR mutations. A phase III clinical trial evaluating first-line amivantamab plus galazetinib in combination with osimertinib in EGFR-mutated NSCLC is currently underway (60). In patients with MET amplification pretreated with EGFR-TKIs, the first-generation EGFR-TKI gefitinib in combination with the MET inhibitor capmatinib has demonstrated effectiveness (61). Tepotinib, another MET TKI, showed a higher objective response rate (ORR) in combination with gefitinib in phase Ib/II trials (62). Moreover, the natural product berberine has shown MET-inhibiting ability and synergistic induction of apoptosis with osimertinib, thus overcoming MET-enhanced induction of osimertinib-resistant cancers (63). Preclinical trials with HQP8361 (64) and dictamnine (65), two MET receptor inhibitors, have demonstrated anti-osimertinib resistance properties. Increased MET copy number has also been identified as a common mechanism of resistance to rosatinib, which can be overcome by the MET inhibitor crizotinib (36).

AXL RTK activation is another resistance mechanism. Combining AXL inhibitors with axitinib has shown efficacy in overcoming resistance to axitinib in EGFR-mutant NSCLC (66). Novel AXL inhibitors, including AXL/MET dual inhibitors and AXL-specific antibody-drug conjugates, have exhibited potent effects in overcoming osimertinib resistance in preclinical trials (67, 68).

Additionally, many EGFR-TKI resistance mechanisms involving bypass pathway activation can be addressed through bypass inhibition. *In vitro* studies have shown that the addition of PI3K inhibitors to EGFR-TKIs can overcome resistance, and combining MEK and PI3K inhibitors has proven to be an effective therapeutic strategy for treating NSCLC with acquired resistance to EGFR-TKIs (69). In cases of drug-resistant NSCLC resulting from FGFR gene family amplification, combined inhibition of FGFR and AKT has shown efficacy in FGFR1-overexpressing osimertinib-resistant NSCLC cells (70). Simultaneous inhibition of SFK/FAK and EGFR may also hold promise as a therapeutic strategy (50). RET fusion activates a bypass pathway leading to resistance to EGFR-TKIs, and this pathway can be effectively targeted in the clinic using a selective RET inhibitor (BLU-667) (47). Activation of the insulin-like growth factor 1 receptor (IGF1R) can be addressed through the use of IGF1R inhibitors in combination with octreotide, and clinical trials have been conducted to evaluate their safety and efficacy (71, 72). Furthermore, exon 16 skipping HER2 deletion (HER2D16) leads to osimertinib resistance, and unlike in breast cancer, HER2D16 mediates resistance in NSCLC through a Src-independent mechanism, making it insensitive to Src inhibitors (45). Combining osimertinib with the pan-HER small molecule inhibitor afatinib has shown synergistic potential in overcoming HER2D16 resistance (45). EGFR-TKI resistance due to BIM polymorphism can be circumvented in combination with HDAC Inhibition (73). ROS1 rearrangement caused by EGFR-TKIs can be overcome by combining crizotinib (74, 75).

3) Fourth-generation EGFR-TKIs

Currently, fourth-generation EGFR-TKIs are undergoing clinical investigation, with a primary focus on targeting T790M/C797S drug-resistant mutations. EAI045, the first selective small-molecule variant inhibitor, has shown efficacy in mouse NSCLC models when used in combination with cetuximab. However, its clinical introduction has been hindered by significant adverse effects (76). JBJ-04-125-02 has demonstrated greater durability compared to EAI001 and has shown the ability to slow down C797S resistance either as a monotherapy or in combination with other agents (77). Another promising fourth-generation EGFR-TKI, tQB3804, has shown the ability to overcome resistance mediated by multiple mutations and is currently undergoing phase I clinical trials (78).

4) Antibody-drug conjugates (ADCs)

In addition to the approaches mentioned above, antibody-drug conjugates (ADCs) have emerged as an effective treatment strategy for overcoming drug resistance. Although most of the them target HER family, an increasing number of trials have shown that this ADC has therapeutic effects in patients who are resistant to EGFR-TKIs. Clinical activity of HER3-DXd, an ADC targeting HER3, has been observed independent of the resistance mechanism in a phase I clinical trial involving patients resistant to EGFR-TKIs, suggesting a novel approach for treating EGFR-TKI-resistant NSCLC patients (79).

#### 2.1.3 KRAS: the mechanisms of resistance

KRAS is a small GTPase that exhibits reduced ability to hydrolyze GTP or interact with GTPase-activating protein (GAP) when mutated. This results in KRAS being locked into an active GTP-binding state, promoting cancer cell growth and anti-apoptosis (80). KRAS mutation represents the most prevalent genetic alteration observed in NSCLC (81). The most common KRAS mutations in NSCLC is KRAS(G12C) accounting for 45%, followed by KRAS(G12V) and KRAS(G12D) (82, 83).

Currently, the clinical agents targeting KRAS mutation in NSCLC include AMG 510 (sotorasib) (84) and MRTX849 (adagrasib) (85). These inhibitors covalently bind to cysteine residues in the switch-II pocket, which is generated by the G12C mutation. This binding favors GDP over GTP binding, reducing interactions between mutant KRAS and effector or regulatory molecules.

However, monotherapy with KRAS inhibitors is prone to adaptive resistance for a number of reasons, including the following:

1) RAS target-dependent resistance mechanisms:

The reactivation of the RAS pathway in an adaptive manner is crucial in the emergence of resistance to inhibitors targeting KRAS G12C (86). For instance, increased HER2 copy number leads to the persistence of MAPK pathway signaling and resistance to inhibitors. This resistance can be overcome by targeting SHP2 (87). RTKs such as the ErbB family or FGFR can also maintain KRAS in an activated GTP-bound state via SHP2, attenuating the effects of KRAS(G12C) inhibitors (88, 89). Additionally, amplification of MET can lead to resistance of NSCLC to KRAS(G12C) inhibitors (86). A patient treated with MRTX849 developed polyclonal acquired resistance and reactivation of RAS-MAPK signaling. Some cancer cells develop non-G12C KRAS mutations, resulting in resistance to KRAS inhibitors. For example, a study identified a novel KRAS Y96D mutation that interferes with protein-drug interactions in the switch-II pocket and confers resistance to inhibitors in a KRAS G12C cancer model (90). Acquired oncogenic mutations in KRAS, NRAS, or BRAF have also been observed during G12C inhibitor treatment (91).

2) KRAS target-idependent resistance mechanisms:

Certain cancer cells become independent of KRAS dependence by activating alternative pathways. For instance, BET-regulated YAP1 upregulation was found to contribute to resistance acquisition in KRAS; LKB1 and KRAS; TP53 mutant NSCLC cells (92).

#### 2.1.4 KRAS: the strategies to overcome resistance

Currently, combination therapy is the primary approach to overcome KRAS inhibitor resistance. Dual blockade of MET and KRASG12C has shown significant efficacy in MET-amplified, KRASG12C-mutant NSCLC (86). The MET inhibitor crizotinib restored sensitivity to sotorasib by inhibiting RAS-MEK-ERK and AKT signaling (86). Inhibition of SHP2 reduced the conversion of GDP to GTP-bound KRAS and overcame adaptive resistance to MAPK pathway-targeting drugs, including KRAS(G12C) inhibitors (93). Additionally, the combination of TBK1 and MEK inhibition, along with intermittent BET inhibition, has been shown to overcome resistance to KRAS inhibitors by Kitajima et al (92). Furthermore, in a xenograft mouse model, the combination of the clinical KRAS inhibitors AMG-510 and MRTX849 with carboplatin and palbociclib resulted in significant regression of lung tumors (94, 95).

Feedback reactivation of the adaptive RAS pathway, non-G12C KRAS mutations, and bypass activation are the three major mechanisms underlying KRAS inhibitor resistance. Given these challenges, the use of combination therapy has arisen as a successful approach to treatment. The combined therapy of crizotinib + sotolacib has been employed to address the issue of resistance to sotolacib caused by MET amplification (86). Similarly, for resistance arising from HER2 amplification, the combination of TNO155 + sotorasib has demonstrated promising results (87). Given that feedback reactivation of the adaptive RAS pathway represents a key mechanism driving resistance to KRAS G12C inhibitors, targeted inhibition of SHP2 assumes paramount importance in overcoming KRAS resistance.

#### 2.1.5 ALK: the mechanisms of resistance

ALK is a transmembrane RTK, which belongs to a subfamily within the insulin receptor superfamily (96). ALK is encoded by ALK gene, which is located on the short arm of chromosome 2 (2p23) (97). In the process of oncogenesis, the ALK gene translocates with another partner gene, leading to a fusion oncogene that overexpresses in cancers (98). A minority (3–13%) of NSCLC cases have been demonstrated to exhibit rearrangements in the ALK gene (99).

A series of ALK-TKIs, including crizotinib, ceritinib, alectinib, brigatinib, ensartinib, and lorlatinib, have been approved for ALK-positive patients worldwide. More specifically, crizotinib, ceritinib, and lorlatinib work as ATP-competitive inhibitors (100–102); alectinib and brigatinib can inhibit ALK protein by preventing phosphorylation (103); ensartinib acts by inhibiting ALK fusions engineered to have point mutations (104).

The first-generation ALK inhibitor is crizotinib. Its resistance mechanisms can be categorized into ALK-dependent mechanisms and ALK-independent mechanisms (105). The former is constructed by point mutations, including F1174L, L1152R, S1206Y, 1151Tins, I1171T, D1203N, V1180L, C1156Y, F1164V, G1269A, G1202R, and G1269S (106, 107), as well as gene amplification, which implicated in 9% of the secondary mutations of the ALK gene cases (108). While ALK-independent mechanisms refer to the activation of bypass signaling through the activation of other oncogenes, like EGFR, extracellular signal regulated kinase (ERK), Crk-like adaptor protein etc. Also, the primary resistance of crizotinib, whose RR is about 60%, should not be ignored. The primary resistance is the different variants of the EML4–ALK fusion protein or the false-positive diagnoses of ALK translocation (109).

The second-generation ALK inhibitors, ceritinib, alectinib, and brigatinib, were introduced due to the drug-resistance of the first-generation ALK inhibitor, which show predominant effects on crizotinib-resistant patients (110). Nevertheless, the wide use of second-generation ALK inhibitors can inevitably lead to drug resistance and tumor relapse (111), which can also be divided into ALK-dependent mechanisms and ALK-independent. More than half of the ceritinib-resistant specimens showed secondary mutations, such as G1123S, F1174C/L/V, and G1202R mutations (112). And ALK-independent mechanisms included activating mutation of MEK, the overexpression of p-glycoprotein ABCB1, SRC activation and overexpression of SRC homology 2 domain (SHP2), and activation of IGF-1 R, KIT, and EGFR (112). G1202R, G1206C, G1206Y, E1210K, F1245C, G1269A, G1269S, V1180L, and I1171T/N/S mutations can be relevant to the alectinib-resistance by affecting the binding between the drug and the ALK fusion protein or other ways (112, 113). Some bypass tracks, like C-MET activation, Coactivation of MET and Proto-oncogene tyrosine-protein kinase Src (SRC), Yes-associated protein 1 (YAP 1), Amphiregulin, the overexpression of ATP-binding cassette subfamily C member 11 (ABCC11), Transformation to SCLC, and EMT, can cause alectinib-resistance as well (112, 114–118). It is also reported that the brigatinib-resistance may be associated with the mutation of G1202R, I1171T, I1171N, V1180L, G1202R, T1151M, L1196M, E1408V, and ALK amplification (119–121), and some compound mutations including E1210K+S1206C and E1210K+D1203N (110). We can only make a conclusion that the ALK-dependent mechanisms of brigatinib are still unclear. The brigatinib-resistance is also caused by MTOR T1834-T1837del, JAK3R948C mutation, CDKN2A/B loss, and NFE2L2E79Q mutation, and MET amplification, mutation of BRAF-V600E, and KRAS -G12D (112).

Lorlatinib, a third-generation ALK/ROS1-TKI, acts as an ATP-competitive macrocyclic. However, just like the first- and second-generation ALK inhibitors, acquired lorlatinib-resistance still existed in nearly all patients (112). It has been reported that compound mutations can be identified among more than one-third of lorlatinib-resistance cases. And the ALK-independent mechanisms included activation of EGFR, TP53, NRAS-G12D, and MAP3K1 mutations, neurofibromatosis type 2 (NF2) loss-of-function mutations, EMT, and transformation to neuroendocrine carcinoma (105, 110, 114, 122, 123).

#### 2.1.6 ALK: the strategies to overcome resistance

By activating bypass signaling to deal with ALK TKI resistance, the combination with other inhibitors targeting different kinases can be a beneficial addition as well. For example, the joint utilization of the MEK inhibitor selumetinib and ceritinib has been proven to show significant therapy effect on MAP2K1K57N activation mutation of MEK NSCLC cell line, and dual blockage of ALK/MEK can overcome even delay the ALK-TKL resistance (105). Gilteritinib effectively counteracts the development of resistance to lorlatinib in cancer cases with ALK rearrangements (124).

In addition, a research has demonstrated good responses to immunotherapy with immune checkpoint inhibitors, namely nivolumab, pembrolizumab, and atezolizumab, in advanced NSCLC (125). In addition, ALK-TKIs and immune checkpoint inhibitors are also used in combination therapy, including crizotinib with nivolumab, or ipilimumab, or pembrolizumab; alectinib with atezolizumab; ceritinib with nivolumab and lorlatinib with avelumab (105).

#### 2.1.7 ROS1: the mechanisms of resistance

The ROS1 gene, encoding a RTKRTK of the insulin receptor superfamily, shares significant homology with ALK (126). When the ROS1 gene undergoes a genetic alteration, such as a fusion with another gene, it can lead to the formation of a fusion protein with abnormal signaling properties. This fusion protein is constitutively activated, resulting in uncontrolled cell growth and division, which is a hallmark of cancer (127). ROS1-dependent cancers are a specific group of tumors that rely on the abnormal activity of the ROS1 fusion protein for their growth and survival. These cancers include certain types of NSCLC (126, 128), glioblastoma, cholangiocarcinoma, and others. In recent years, targeted therapeutic agents like lorlatinib and crizotinib, have emerged as promising treatment options for ROS1-positive NSCLC patients. Despite the initial success of ROS1-targeted therapies, the development of resistance remains a challenge. Several resistance mechanisms have been identified, including:

1) ROS1 target-dependent resistance mechanisms:

ROS1 kinase domain mutations may include gene fusions or mutations that result in constitutive activation of ROS1 kinase activity. For example, ROS1 gene fusions with other genes, such as CD74, SLC34A2, and EZR (129, 130), have been identified in NSCLC. These fusions lead to the overexpression of ROS1 and the constitutive activation of its kinase domain, promoting uncontrolled cell growth and tumor formation. ROS1 kinase domain mutations are detected in more than 30% of crizotinib-resistant and nearly 50% of lorlatinib-resistant cases (131). Lorlatinib does not possess sufficient efficacy against the ROS1G2032R mutation, which is known to be resistant to crizotinib. Furthermore, lorlatinib also lacks potency against the ROS1L2086F mutation (131). Consequences of several ROS1 mutations include both functional and steric effects. ROS1G2032R causes a steric clash with the piperidine ring of crizotinib, thereby impeding its effective binding as a TKI (132). Similarly to the ALKL1196M resistance substitution, ROS1L1951R also engenders steric hindrance against crizotinib binding (133). In a comparable manner, the αC helix undergoes a positional change induced by ROS1S1986Y/F, which further obstructs crizotinib binding (134). NSCLC induced by a mutation in CD74–ROS1 develops resistance to crizotinib (135). Moreover, experimental evidence demonstrates that ROS1G2032R induces epithelial-mesenchymal transition and amplifies the migratory and invasive capacities of ROS1 fusion-driven cancer cells through the upregulation of Twist1 (136).

2) ROS1 target-dependent resistance mechanisms:

However, in addition to the genetic alterations within the ROS1 gene itself, there are extrinsic mechanisms that can affect the activity and response of ROS1-dependent cancers. Activation of bypass signaling pathways is involved in this process, such as KRAS, NRAS, EGFR, HER2, KIT, BRAF, and MEK (134, 137–139), have been implicated. In the clinical setting, mutations in KRASG12D and BRAFV600E have emerged during crizotinib treatment (140).

#### 2.1.8 ROS1: the strategies to overcome resistance

Researchers have made significant progress in addressing ROS1-targeted therapy resistance. Several strategies and treatment options have been explored.

Next-generation ROS1 inhibitors are being developing. Currently undergoing clinical evaluation in ALK and ROS1 fusion-positive NSCLC cancers, PF-06463922, a next-generation ROS1/ALK small-molecule inhibitor, exhibits potent inhibition of various oncogenic ROS1 fusion variants and selective activity against a wide range of kinases (133). Repotrectinib (TPX-0005) shows effectiveness against acquired solvent-front mutations in ROS1, NTRK1–3, and ALK, making it a promising treatment for patients with ROS1-, NTRK1–3-, or ALK-rearranged malignancies who have progressed on prior TKIs (141).

Combination therapy is also one of the potentially effective treatment approaches. Combining ROS1-targeted agents with other targeted therapies or immunotherapies has been investigated to enhance treatment efficacy and overcome resistance. For example, combining ROS1 inhibitors with MEK inhibitors or immune checkpoint inhibitors has shown potential in preclinical and clinical studies (142, 143).

Furthermore, ongoing research focuses on identifying novel targets and resistance mechanisms to inform the development of new treatment strategies. For instance, targeting bypass or compensatory signaling pathways, such as MAPK, MET, or AXL, may provide additional avenues to combat resistance (144–146).

#### 2.1.9 BRAF: the mechanisms of resistance

BRAF, a member of the Raf kinase family, plays a crucial role in cell growth, proliferation, and differentiation through the MAPK pathway (147). BRAF mutations can be classified into V600 and non-V600 mutations. V600E mutations are the most common type of BRAF mutation in NSCLC (55%), followed by G469A (35%), D594G (10%), and others (148, 149). The V600E mutation leads to an overload of the MAPK pathway and reduces the activation of apoptotic mechanisms regulated by BAD and cysteine cascade events (150).

In monotherapy studies of NSCLC patients, darafenib demonstrated activity in a prospective study of NSCLC with BRAF mutations (151). Targeted agents for advanced BRAF-mutated NSCLC include vemurafenib, dabrafenib, or sorafenib (152). *In vitro* studies have confirmed the effectiveness of the MEK-TKI trametinib alone in BRAF V600E-mutated NSCLC cell lines (153). However, targeted therapy is currently not applicable to patients with non-V600 BRAF mutations (154). BRAF inhibitors effectively inhibit V600 mutation monomers, but in non-V600 mutation dimers, the drug can only bind to one of the sites, significantly reducing affinity for the second site and downstream ERK inhibition. Consequently, BRAF non-V600 mutant tumors are theoretically insensitive to BRAF inhibitors, as supported by *in vitro* experiments (155). Certain EGFR-TKIs, such as gefitinib, afatinib, and osimertinib, have been shown to directly inhibit the G469V BRAF mutant, suppressing NSCLC cell growth *in vitro (* 154).

The mechanisms of BRAF inhibitor resistance in NSCLC have not been fully elucidated. For BRAF V600E mutant NSCLC, acquired resistance after dabrafenib monotherapy is caused by oncogenic KRAS mutations and subsequent sustained BRAF non-dependent MEK activation (156). Kim et al. (157) found that treatment of a dalafenib-resistant (GSR) cell line (V600E NSCLC) with dalafenib resulted in upregulation of EGFR, activation of the EGFR-RAS-CRAF pathway, and sustained activation of ERK1/2, enhancing EGFR-mediated RAS activity. In BRAF non-V600E mutant NSCLC, a study demonstrated that single-agent trametinib upregulates the AKT signaling pathway in this cell line, indicating resistance to single-agent MEK inhibitors (158). Additionally, BRAF non-V600E mutations often coexist with RAS mutations, which may contribute to drug resistance (159). Acquired BRAF mutations can also occur in tumors with EGFR mutations and are suspected to be a mechanism of resistance to the EGFR-TKI osimertinib (160).

#### 2.1.10 BRAF: the strategies to overcome resistance

Although BRAF inhibitors have shown efficacy in various cancers, most patients eventually develop resistance. Combination therapies have been found to be more effective than monotherapy and can delay or overcome resistance. In BRAF V600E-mutated NSCLC, the combination of dabrafenib and trametinib targeting BRAF and MEK demonstrated promising response rates compared to dabrafenib monotherapy (161). However, this therapy is not effective in patients with non-V600 mutated tumors. Limited studies exist on drug resistance strategies for NSCLC with BRAF non-V600E mutations. When trametinib was combined with vemurafenib to treat cell lines, the AKT pathway was not upregulated, suggesting that combination therapy may be beneficial in overcoming drug resistance (158). Chen et al. (162) found that atypical BRAF mutant cells were resistant to the BRAF inhibitor vemurafenib but sensitive to the RAF dimer inhibitor LY3009120, presenting a potential therapeutic option. For acquired BRAF mutations caused by EGFR mutations, drug combinations effectively alleviate BRAF mutation-induced osimertinib resistance. An *in vitro* study revealed that osimertinib resistance caused by acquired BRAF G469A mutation could be restored with selumetinib and trametinib, rather than dabrafenib treatment (163). Furthermore, several cases of acquired BRAF V600E mutations have shown that patients respond well to the concurrent combination of dabrafenib, trametinib, and osimertinib (164–167). Li et al. (168) provided clinical evidence that trametinib plus osimertinib is effective in patients with EGFR-mutated NSCLC carrying acquired BRAF p.D594N mutation. Sun et al. (169) demonstrated that concurrent treatment with vemurafenib and osimertinib, targeting both EGFR and BRAF, resulted in regression of BRAF V600E mutation-induced osimertinib resistance.

BRAF V600E NSCLC is a prevalent subtype known for its resistance to BRAF inhibitors, mainly due to the existence of KRAS mutations leading to continuous activation of the MEK pathway regardless of BRAF (156). However, the combination of dabrafenib and trametinib has demonstrated efficacy in alleviating this resistance (161). Notably, the BRAF inhibitor (BRAFi) and MEK inhibitor (MEKi) combination has received approval from the US Food and Drug Administration (FDA) for various cancer types (170, 171). Conversely, resistance mechanisms in NSCLC with BRAF non-V600E mutations are not well studied and require more research. Additionally, BRAF mutation serves as a mechanism of drug resistance in EGFR mutation-induced NSCLC. In this context, the combination of BRAF inhibitors has shown promise in overcoming resistance to osimertinib induced by BRAF mutations (163–169).

### 2.2 Uncommon clinical gene targets

#### 2.2.1 RET

The RET gene encodes a RTKRTK that exerts its oncogenic effects through chromosomal rearrangements generating hybrid proteins (172) as well as mutational activation (173). The proportion of patients with NSCLC who have RET rearrangements is approximately 1%-2% (174–176).

Resistance mechanisms to RET multikinase inhibitors and selective inhibitors can be classified into three main categories:

secondary RET mutations (e.g. RET Gatekeeper mutation V804L/M), acquisition of mutations in genes other than RET, and bypass activation (177). The majority of NSCLC patients with RET fusion have not yet revealed a clear mechanism of drug resistance, preclinical studies have demonstrated that both RET G810S (178) and RET V804 (179, 180) mutations can confer resistance to RET inhibitors in NSCLC. Another avenue of resistance is through MET-mediated bypass activation, which can be overcome by co-inhibition strategies (181, 182). Moreover, RET fusions have been identified as a mechanism of acquired resistance to osimertinib in EGFR mutant NSCLC cases (47). Continued research in this area will provide valuable insights into overcoming resistance and developing effective treatment approaches.

#### 2.2.2 NRAS

The neuroblastoma rat sarcoma virus oncogene homolog (NRAS) is a member of the RAS family, discovered subsequent to the identification of KRAS and HRAS (183). NRAS encodes small GTPases that play a crucial role in regulating cell cycle, proliferation, maturation, and differentiation by transducing signals from membrane-bound RTKs to the nucleus. Mutations in NRAS lead to persistent activation of Ras-GTP, thereby promoting tumorigenesis and metastasis. NRAS mutations are infrequent in NSCLC patients, and there are currently no approved therapies specifically targeting NRAS-mutated NSCLC.

LXH254, a type II pan-RAF inhibitor with high selectivity for BRAF and CRAF, has exhibited antitumor activity in preclinical models of NRAS mutations (184). Park et al. reported that human NRAS-mutant NSCLC cells display moderate sensitivity to PAN-RAF inhibitors, and dual-targeted inhibition of PLK1 and PAN-RAF using Volasertib in combination with LXH254 is more effective in inhibiting long-term cell survival compared to LXH254 alone. This combination therapy may serve as a valuable source of inspiration and guidance for clinical treatment (185).

Nonetheless, substantial gaps remain in the research concerning resistance to NRAS-targeted therapy in NSCLC treatment. It has been demonstrated that miR-145-5p enhances the sensitivity of acquired gefitinib-resistant cells to gefitinib by suppressing NRAS and MEST expression (186). To advance our understanding of NRAS-associated resistance mechanisms in NSCLC treatment, further rigorous investigations are warranted.

#### 2.2.3 PIK3CA

Alterations in the phosphatidylinositol 3-kinase (PI3K) pathway are prevalent in cancer (187). Downstream signaling pathways of PI3K involve AKT and mTOR, while phosphatidylinositol 4,5-bisphosphate 3-kinase catalytic subunit alpha (PIK3CA), encoding the catalytic subunit p110α of the PI3K complex, is frequently mutated and amplified in various cancer types. Genetic alterations in PIK3CA contribute to oncogenesis in NSCLC and activate mutations in both EGFR and KRAS (188). The incidence of PIK3CA mutations in NSCLC patients is approximately 3.7% (189).

Currently, there is no approved targeted therapy specifically for PIK3CA. The most promising candidate is Alpelisib (BYL719), a potent and selective PI3Kα inhibitor currently undergoing phase II clinical studies for the treatment of NSCLC. Regarding the resistance of PIK3CA-targeted therapy in NSCLC, an *in vitro* study involving a panel of cancer cell lines revealed that BRAF and phosphatase and tensin homolog (PTEN) mutations, along with concurrent PIK3CA and KRAS mutations, have the potential to render cells insensitive to BYL719 treatment (190). A clinical case report on a NSCLC patient with resistance to alpelisib identified a MET exon 14 skipping mutation, possibly serving as a resistance mechanism to alpelisib (191). Previous studies have also implicated MET exon 14 skipping mutations as mechanisms of resistance to TKI inhibitors (192). Additionally, miR-10a enhances the resistance of circulating tumor cells (CTCs) to cisplatin and inhibits the PI3K/Akt pathway by targeting PIK3CA, providing a novel therapeutic target for NSCLC treatment (193). Inhibiting EZH2 enhances the sensitivity of PIK3CA-driven NSCLC to PI3K inhibition (194). Limited research exists on specific therapeutic strategies for overcoming treatment resistance induced by PIK3CA mutations in NSCLC. However, studies on breast cancer offer insights that could be applicable to NSCLC. PDK-1 signaling is known to activate the mTOR complex without activating AKT (195), and IGF-1 and other growth factors can also activate mTOR signaling, leading to resistance to PI3K inhibitors (196). Therefore, combining PI3K, AKT, mTOR, or CDK 4/6 inhibitors can enhance therapeutic efficacy. Furthermore, while PI3K inhibitors reduce AKT signaling, AKT inhibitors can slow down the development of resistance to PI3K inhibitors in breast cancer cells (197). Synergistic inhibition of CDK4/6 and PI3K inhibitors may overcome resistance to PI3K inhibitor monotherapy (198).

It is noteworthy that the majority of studies investigating PIK3CA resistance have primarily focused on breast cancer cells, while there remains a paucity of research specifically examining resistance mechanisms in NSCLC. Therefore, further investigations into the molecular mechanisms underlying PIK3CA resistance in NSCLC are warranted to enhance our understanding and inform the development of effective therapeutic strategies.

#### 2.2.4 HER2

HER2, a member of the HER family along with HER1 (also known as EGFR), HER3, and HER4, forms heterodimers with other HER family receptors or homodimers when highly expressed (199). These interactions subsequently lead to tyrosine kinase phosphorylation and activation of downstream tumorigenic signaling pathways, including RAS-RAF-MEK-ERK and PI3K-AKT. HER2 alterations, which are widely acknowledged as a key factor in the development of various types of solid tumors, primarily consist of HER2 mutation, HER2 amplification, and HER2 overexpression. The incidence rates of these alterations in NSCLC are approximately 1%-6.7%, 2%-22%, and 7.7%-23%, respectively (200–204).

Early targeted therapy for HER2 mutations in NSCLC patients primarily involved pan-HER-TKIs such as afatinib (205–208), daclatinib (209), neratinib (210), poziotinib (211), and pyrotinib. However, the results of these prospective studies with small sample sizes were unsatisfactory (206, 208, 210, 212). The antibody-drug conjugate T-DM1 has demonstrated favorable short-term efficacy in advanced NSCLC with HER2 mutations, but response duration is limited (213, 214). A relatively new anti-HER2 ADC, T-DXd, comprising the topoisomerase I inhibitor deruxtecan, trastuzumab, and cleavable linkers, has shown promising results. The FDA recently approved a 5.4 mg/kg dose of T-DXd based on data from the clinical studies DESTINY-Lung01 (215) and DESTINY-Lung02 (216) for patients with advanced and metastatic HER2-mutated NSCLC who have received prior systemic therapy. A retrospective analysis of 101 patients with advanced NSCLC harboring HER2 mutations and treated with trastuzumab showed higher overall efficacy when combined with chemotherapy compared to chemotherapy alone (217). *In vitro* experiments have demonstrated that T-DM1 can overcome resistance to gefitinib and osimertinib (218). Another study involving 9 patients with advanced NSCLC and HER2 mutations reported higher efficacy with trastuzumab in combination with chemotherapy compared to afatinib treatment (219). T-DM1 monotherapy has also shown activity in patients with HER2 amplification and concurrent EGFR mutations (213). Furthermore, preclinical models with HER2 amplification have exhibited significant tumor shrinkage when treated with a combination of T-DM1 and the pan-HER inhibitor neratinib, similar to trastuzumab-duocarmycin, another HER2-targeted ADC (213).

The role of HER2 amplification or overexpression as a driver in primary NSCLC remains unclear and requires further thorough investigation. Current research suggests that HER2 amplification is a mechanism by which various types of NSCLC develop drug resistance. HER2 amplification leads to resistance to EGFR-TKIs in NSCLC (30), and HER2 mutations exhibit similar effects (220), with HER2D16 leading to osimertinib resistance via the Src non-dependent pathway (221). In addition, HER2 amplification also contributes to afatinib resistance in HER2-mutated NSCLC (222) and ALK-TKI resistance in ALK-rearranged NSCLC (223). Furthermore, HER2 amplification can cause acquired resistance to MET inhibitors during crizotinib treatment (221) and resistance in KRASG12C NSCLC, which can be overcome by co-targeting SHP2 (87).Current research indicates that HER2 amplification serves as a prominent mechanism underlying the development of drug resistance in various lung cancer types. This resistance extends to EGFR-TKI (30), HER2 self-mutation inhibitors (222), ALK-TKI (223), MET inhibitors (221), and KRAS G12C inhibitors (87). Notably, afatinib, a HER2-targeted inhibitor, has demonstrated limited efficacy in NSCLC patients, failing to meet the anticipated disease control outcomes (206). This lack of response could be attributed to the inherent resistance of HER2 mutants to pan HER TKIs. Moreover, the activation of YES1 leads to the emergence of resistance to neratinib in breast and lung cancers that have HER2 amplification (224).

While studies investigating the mechanisms of resistance to ADCs have primarily focused on breast cancer cells (225, 226), there remains a significant gap in our understanding of these mechanisms within the context of NSCLC cells. Further investigation is required to elucidate the specific resistance mechanisms operating in NSCLC cells, thus enabling the development of effective therapeutic strategies.

#### 2.2.5 c-MET

c-MET is a member of the tyrosine kinase receptor superfamily and is encoded by the MET proto-oncogene. Structurally, it is a transmembrane tyrosine kinase receptor protein comprised of a heterodimer consisting of a 45 kDa extracellular α subunit and a 145 kDa transmembrane β subunit (227). The extracellular regions of both subunits serve as the site for ligand recognition, while the intracellular region possesses tyrosine kinase activity. This activity is responsible for activating tyrosine kinase upon ligand binding and subsequently initiating downstream cascade signals (228). In patients with NSCLC, MET exon 14 jump mutations are present in around 5.6% of patients (229). On the other hand, MET amplification has been observed in a broader range of 2% to 20% in various studies. This variation may be attributed to disparities in the definition of amplification levels and the methods used for detection (230).

MET inhibitors can be categorized into three main classes: small molecule MET receptor inhibitors (e.g., crizotinib, tivantinib, savolitinib, tepotinib, cabozantinib, and foretinib), MET receptor monoclonal antibodies (e.g., onartuzumab, emibetuzumab), and antibodies targeting their ligands HGF (e.g., ficlatuzumab and rilotumumab) (231). Of these, Crizotinib has gained FDA approval for treating ALK-positive patients with advanced NSCLC. It is an ATP-competitive, non-selective inhibitor with multiple targets, including c-Met and ALK (232). Additionally, Merck’s TepMetko (tepotinib) has obtained approval in Japan for treating patients with advanced NSCLC harboring MET exon 14 skipping mutations. This represents the world’s first approved single-target inhibitor for c-MET.

Resistance mechanisms to inhibitors targeting MET exon 14 skipping mutations can be categorized into primary and secondary resistance. Primary resistance primarily arises from mutations and alterations in exon splice sites (233, 234), whereas secondary resistance is often associated with HER2 amplification (221). MET amplification commonly contributes to resistance development in various other NSCLC treatments, particularly in the context of acquired resistance to EGFR-TKI (235, 236). Moreover, modifications in the signaling molecules’ functionality have also been identified as contributing factors in the emergence of drug resistance.

Overcoming resistance to targeted therapies in NSCLC is a significant challenge, but there are several promising strategies that researchers are exploring. Clinical trials and ongoing research efforts are essential to further explore and optimize these approaches for overcoming resistance to targeted therapies in NSCLC. Above all, In the recent 5 years, the promising strategies for overcoming the resistance to targeted therapies in NSCLC are summarized in **Table 1**.

**Table 1:** The promising strategies for overcoming the resistance to targeted therapies in NSCLC in recent 5 years.

Resistant to	Resistance Mechanisms	Target	Promising Strategies	Reference
osimertinib	C797S	EGFR L858R	Erlotinib + Afatinib	(25, 53)
All EGFR-TKIs	trans-C797S/T790M	EGFR	First generation TKIs + third generation TKIs	(29)
osimertinib	G724S	EGFR	Afatinib	(40)
Osimertinib	HER2D16	EGFR	Osimertinib + Afatinib	(45)
osimertinib	L718Q	EGFR L858R	Afatinib	(53)
osimertinib	MET amplification	EGFR	amivantamab + lazertinib	(60)
osimertinib	MET amplification	EGFR	HQP8361 (64)/berberine (63) (MET kinase inhibitor)+ Osimertinib	(63, 64)
Gefitinib/osimertinib.	MET dysregulation	EGFR	Dictamnine (c-Met inhibitor)	(65)
Osimertinib/dacomitinib	AXL overexpressing	EGFR	ONO-7475(AXL Inhibitor) + osimertinib	(68)
Osimertinib	FGFR1 overexpressing	EGFR	FGFR inhibitor + AKT inhibitor	(70)
gefitinib	BIM deletion polymorphism	EGFR	Vorinostat (HDAC inhibitor)	(73)
EGFR-TKIs	ROS1 rearrangement	EGFR	crizotinib	(74, 75)
EGFR-TKIs	HER3 augmentation	EGFR	HER3-DXd	(79)
sotorasib	MET amplification:	KRAS	Crizotinib+ sotorasib	(86)
sotorasib	HER2 amplification	KRAS G12C		(87)
TBK1/MEK inhibition	BET-regulated YAP1 upregulation	KRAS	TBK1i(Compound 1) + MEKi(selumetinib) + intermittent BETi(GS-626510)	(92)
Lorlatinib	the activation of bypass tracks	EGFR, TP53, NRAS-G12D	combination with other inhibitors	(105, 110, 114, 122)
Crizotinib	point mutations, fusion gene amplification	F1174L, L1152R, S1206Y, 1151Tins, I1171T, D1203N, V1180L, C1156Y, F1164V, G1269A, G1202R, G1269S.	arrangement of the usage of various generations of drugs	(106, 107)
Crizotinib	the EML4–ALK fusion protein/false-positive of ALK translocation			(109)
Brigatinib	Combined mutation	E1210K+S1206C and E1210K+D1203N	combination with other inhibitors	(110)
Crizotinib	the activation of bypass tracks	EGFR, extracellular signal regulated kinase (ERK), Crk-like adaptor protein	combination with other inhibitors	(111)
Alectinib	Mutations	G1202R, G1206C, G1206Y, E1210K, F1245C, G1269A, G1269S, V1180L, and I1171T/N/S mutations	arrangement of the usage of various generations of drugs	(112, 113)
Ceritinib	Mutations	G1123S, F1174C/L/V, and G1202R mutation	arrangement of the usage of various generations of drugs	(112)
Brigatinib	the activation of bypass tracks	MTOR T1834-T1837del, JAK3R948C mutation, CDKN2A/B loss, and NFE2L2E79Q mutation, and MET amplification, mutation of BRAF-V600E, and KRAS -G12D	combination with other inhibitors	(112)
Ceritinib	the activation of bypass tracks	C-MET activation, Coactivation of MET and Proto-oncogene tyrosine-protein kinase Src (SRC), Yes-associated protein 1 (YAP 1), Amphiregulin, the overexpression of ATP-binding cassette subfamily C member 11 (ABCC11), Transformation to SCLC, and EMT	combination with other inhibitors	(112)
Ensartinib	Mutations	G1269A, G1202R, and E1210K	arrangement of the usage of various generations of drugs	(237)
Alectinib	the activation of bypass tracks	C-MET activation, SRC, YAP 1, ABCC11, Transformation to SCLC, and EMT	combination with other inhibitors	(114–118)
Brigatinib	Mutations and gene tracks	G1202R, I1171T, I1171N, V1180L, G1202R, T1151M, L1196M, E1408V, and ALK amplification	arrangement of the usage of various generations of drugs	(119–121)
Lorlatinib	Mutations	MAP3K1, NF2 loss-of-function mutations, EMT, transformation to neuroendocrine carcinoma	arrangement of the usage of various generations of drugs	(123)
lorlatinib	ROS1 mutation	ROS1 G2032R, ROS1 S1986F/L2000V, ROS1L2086F	alternative ROS1 inhibitors (alectinib, brigatinib, or ceritinib, gilteritinib)	(124)
ceritinib	ROS1 mutation	ROS1G2032R	alternative ROS1 inhibitors (Repotrectinib)	(131)
brigatinib	ROS1 mutation	ROS1G2032R	alternative ROS1 inhibitors (Repotrectinib)	(131)
entrectinib	ROS1 mutation	ROS1G2032R, ROS1 L2086F	alternative ROS1 inhibitors (Repotrectinib)	(131, 141)
crizotinib	ROS1 mutation	ROS1 G2032R, ROS1 L2086F	alternative ROS1 inhibitors (Repotrectinib)	(135)
dabrafenib	MEK Continuous activation	BRAF V600E	dabrafenib + trametinib	(157, 161)
trametinib	AKT Signaling pathway up-regulation	BRAF G469A	Trametinib + Vemurafenib	(158)
Osimertinib	BRAF V600E mutation	EGFR	dabrafenib + trametinib + osimertinib	(164–167)
Osimertinib	BRAF p.D594N mutation	EGFR	Trametinib + oxitinib	(168)
Osimertinib	BRAF V600E mutation	EGFR	Vemurafenib + osimertinib	(169)
BLU-667, LOXO-292	solvent front mutation KIF5B-RET G810R	RET G810S	TPX-0046	(178)
Cabozantinib, vandetanib	–	RET V804	Pralsetinib, selpercatinib	(179, 180)
LXH254	–	NRAS	Volasertib + LXH254	(185)
copanlisib	–	PIK3CA	Copanlisib + Tamesemes	(194)
Neratinib	YES1 acceleration	HER2	Dasatinib + Neratinib	(224)

## 3 The mechanisms of immunotherapeutic resistance in NSCLC

### 3.1 Immune checkpoint inhibitors

#### 3.1.1 The definition of immune checkpoint inhibitors resistance

Targeting the PD-L1/PD-1 axis and cytotoxic T-lymphocyte-associated antigen 4 (CTLA-4) with ICIs has shown significant improvements in survival outcomes for patients with NSCLC (238). Several studies have demonstrated that both single-agent and combination ICI therapies have significantly enhanced clinical efficacy endpoints in locally advanced, metastatic NSCLC and extensive-stage small-cell lung cancer patients without EGFR or ALK gene mutations (239). Specifically, for advanced NSCLC patients, ICI treatment can provide long-term benefits, with median response durations ranging from 12 to 25 months, particularly in those with PD-L1 expression ≥50% (240).

Despite the durable benefits indicating the establishment of long-term immune memory, some patients who initially responded to ICI therapy eventually experienced relapse and acquired resistance, leading to clinical and biological immune treatment failure (241, 242). Primary and acquired resistance have emerged as a significant challenge in further improving the prognosis of patients with advanced or metastatic lung cancer (243). Primary resistance is characterized by disease progression within a period of at least 6 weeks (two cycles) but not exceeding 6 months of ICI treatment (244). Acquired resistance, on the other hand, refers to disease progression occurring after at least 6 months of clinical benefit, including objective response or stable disease lasting for at least 6 months (245). Definitions of post-ICI treatment resistance encompass both toxicity-related and unrelated resistance, including resistance occurring after completion of the planned treatment regimen (246). This definition is also applicable to the investigation of adjuvant/neoadjuvant immunotherapy in NSCLC (247). Studies have indicated that the binding of anti-PD-1 receptors decreases after the last dose of treatment for a period of 2-3 months. Therefore, if a patient who previously benefited from ICI therapy experiences disease progression within 12 weeks of the last dose, it is classified as acquired resistance. In such cases, restarting ICI treatment may offer potential benefits (248). On the other hand, patients who received ICI treatment but showed no initial benefit and experience disease progression after stopping treatment for any reason are classified as having primary resistance. However, a recent expert group has suggested that all relapses occurring after initial objective response (excluding stable disease) should be considered acquired resistance, regardless of its timing (249). The concepts of primary and acquired resistance to ICI treatment have evolved from the understanding of resistance in other anti-tumor therapies, such as chemotherapy and tyrosine kinase inhibitors. However, unlike the clear definition of resistance in those therapies, a unified definition for ICI immune resistance patterns has not been established (250). Currently, the most influential concept is proposed by Society for Immunotherapy of Cancer (SITC), which identifies three distinct resistance patterns to anti-PD-1/PD-L1 therapy: primary resistance, acquired resistance, and progression on or after therapy discontinuation (251).

The tumor microenvironment (TME) plays a crucial role in the immune response to ICI treatment (243). Furthemore, the TME evolves based on different embryonic cell genetic backgrounds and tumor genetic compositions (249). In addition, therapeutic interventions such as radiotherapy, surgery, chemotherapy, and immunotherapy, along with clinical and disease characteristics, can influence the TME, potentially affecting the efficacy of single- or combined ICI treatment. Considering different disease progression times and patterns, immune resistance characteristics in NSCLC patients can be categorized into two groups: 1) early progression, including fast progression (FP) or hyper-progressive disease (HPD); 2) late progression after initial treatment benefit (249). These distinct progression times and patterns underscore the immune resistance process observed in diverse patient populations receiving ICI treatment. However, due to the complex nature of biology and the rapid integration of immunotherapy in clinical practice, a unified approach to prevent, define, and manage immune resistance has not yet been established, necessitating further research.

#### 3.1.2 The mechanisms underlying ICI resistance

**Figure 2:**
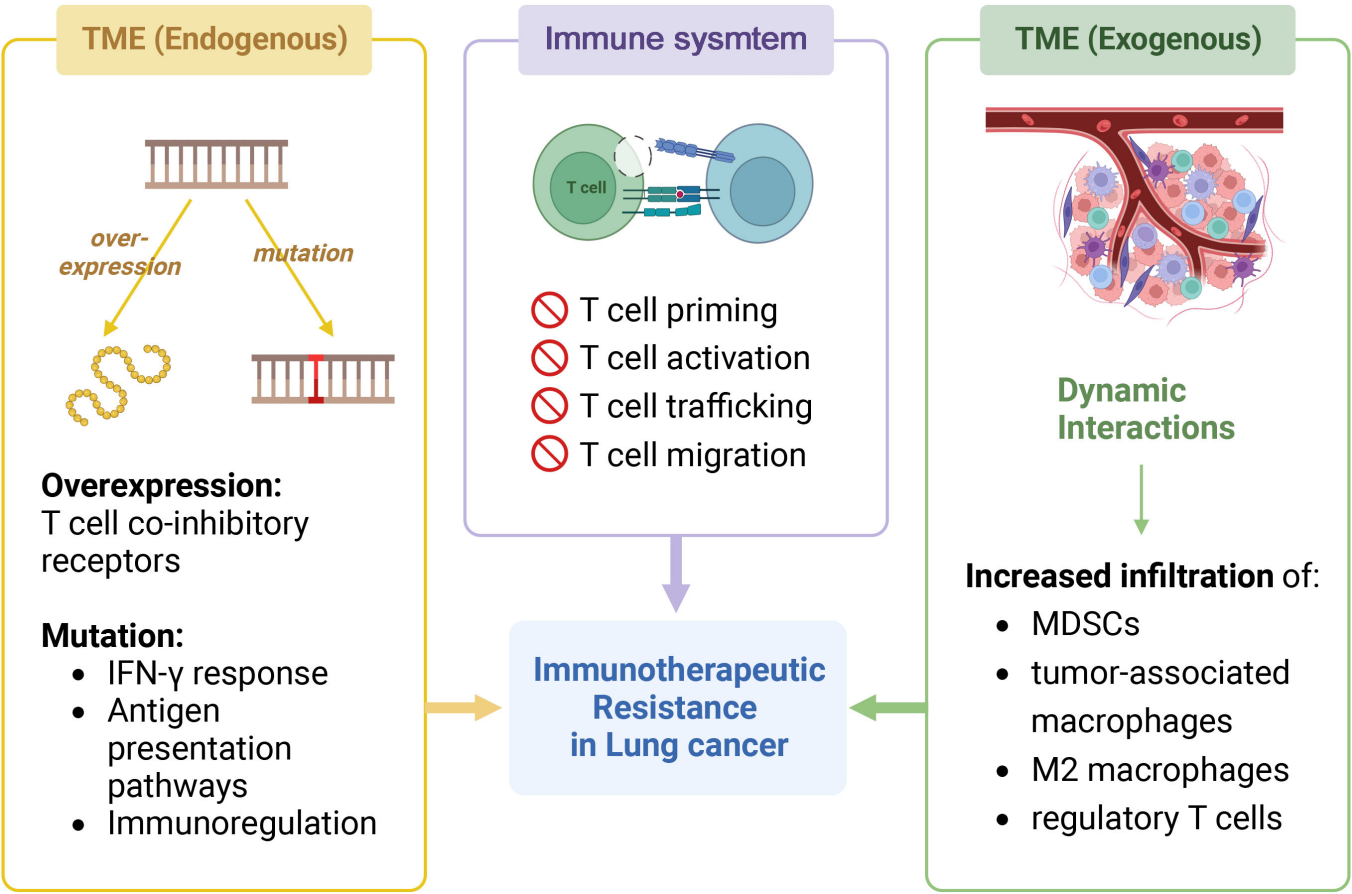
Immunotherapeutic resistance in NSCLC. The image shows the mechanisms that generate resistance in immunotherapy for NSCLC. It can be classified into two parts: immune system and tumor microenvironment (TME), the latter can be further divided into endogenous and exogenous. In immune system, impaired antigen presentation leads to the failure to activate normal immune responses such as T cell priming, activation, trafficking, and migration. In addition, endogenous alterations in the tumor microenvironment leads to the development of drug resistance, including gene overexpression and gene mutation. The former includes T cell co-inhibitory receptors on immunosuppressive cells, and the latter includes genes involved in IFqN-γ response, antigen presentation pathways, and immunoregulation. Exogenous factors such as dynamic interactions within the TME lead to increased infiltration of myeloid-derived suppressor cells (MDSCs), tumor-associated macrophages, M2 macrophages, and tumor-associated macrophages. Created with BioRender.com.

Resistance to ICI is caused by immune system antigen presentation impairment and the TME. The former prevents T cell priming, activation, trafficking, and migration, and the latter can increase resistance by overexpressing T cell co-inhibitory receptors and immunosuppressive cells (244), which can be divided into endogenous and exogenous mechanisms (**Figure 2**).

The endogenous mechanisms for TME resistance can be categorized as follows:

1) Constitutive or acquired mutations in TME genes reduce antigen expression and recognition, preventing T cell activation (252).
2) Mutations in genes relevant to IFN-γ response and antigen presentation, such as antigen processing machinery, signaling transducers, and transcription activators, can disrupt the expression of co-inhibitory signaling and major histocompatibility complex (MHC) molecules (253). Defects in IFN signaling, regardless of MHC-I levels, are the main cause of impaired anti-tumor responses (254).
3) Immunoregulation gene mutations can also affect carcinogenic pathways and the immunosuppressive TME (255). Deficit of β-2 microglobulin can affect MHC-I molecule expression, affecting T cell antigen recognition potential and reducing cytotoxic activity (254). Thus, IL-2 agonists or other non-MHC-I strategies are needed to activate CD8 and NK cells (254).

Exogenous TME factors also matter (254). Dynamic interactions in the TME disrupt immunosuppression, pro-inflammatory cytokines, and mediators like co-stimulatory and co-inhibitory signals, increasing myeloid-derived suppressor cells (MDSCs), tumor-associated, M2 macrophage, and regulatory T cell infiltration (249). Hypoxia increases VEGF expression, which mobilizes and infiltrates MDSCs into tumor sites (256).

However, acquired resistance mechanisms are linked to cancer cell and TME adaptive changes during immune therapy. These changes can be caused by epigenetic regulation or protein translation modification (257). Clonal evolution of cancer cells can also cause acquired resistance by impairing anti-tumor immune responses through gene mutations (258). Furthermore, resistance patterns affect treatment strategy selection (256). Enhanced adjuvant therapy can restore immune sensitivity in acquired resistance patients, allowing immunotherapy (259). Additionally, patient selection criteria and immune resistance patterns may affect research results (259).

### 3.2 Cancer vaccines

Cancer vaccines have emerged as a vital tool in NSCLC treatment, offering potential solutions to challenges associated with conventional therapies and improved patient outcomes. Firstly, cancer vaccines specifically target tumor antigens, focusing the immune response on cancer cells while minimizing harm to healthy tissues, potentially reducing side effects compared to non-specific treatments (260). Secondly, by activating the immune system, cancer vaccines stimulate innate and adaptive immune responses, leading to systemic immune memory and long-term protection against cancer recurrence (261). Personalization is another advantage, as vaccines can be tailored to individual tumor characteristics, enhancing treatment efficacy based on specific antigens or mutations (262). Additionally, the versatility of cancer vaccines allows for combination strategies with other therapies, such as immune checkpoint inhibitors or targeted therapies, to enhance overall immune responses and overcome resistance mechanisms (263). Lastly, cancer vaccines can be applied throughout the various stages of NSCLC, serving as adjuvant therapy post-surgery or as a therapeutic option for metastatic disease (264).

All current investigations into lung cancer vaccines are focused on NSCLC, primarily in participants with advanced disease. Notable vaccines under study for NSCLC include Belagenpumatucel-L (Lucanix), which stimulates the production of TGF-β2 to induce cancer cell death. Phase 2 and 3 trial results indicated that belagenpumatucel-L improved survival rates of NSCLC patients (265, 266). Another vaccine, Stimuvax or Tecemotide, triggers an immune response against mucin 1, a protein overexpressed in NSCLC (267). While a 2011 study showed some positive results, they were not particularly strong (268–270). In contrast, MAGE, the initial cancer/testis antigen (CTA) discovered, exhibits discernible expression in approximately 30%-50% of NSCLC patients, particularly in cases of squamous cell carcinoma (LUSC) (271). The safety of a recombinant adjuvanted MAGE-A3 was assessed in patients with resected MAGE-A3 positive NSCLC through the phase III MAGRIT study. However, the final findings of the study did not demonstrate a superior disease-free survival (DFS) in the MAGE-A3 treated group compared to the placebo control group (60.5 months vs. 57.9 months, respectively) (272). CIMAvax-EGF, a protein-specific vaccine intended to inhibit the EGFR, a protein excessively expressed on NSCLC cells, has exhibited both safety and efficacy in several clinical trials conducted within Cuba (273). In a randomized controlled phase II study encompassing 80 patients with stage IIIB/IV NSCLC who had previously undergone first-line chemotherapy, CIMAvax-EGF demonstrated both safety and immunogenicity (274). Racotumomab prompts antigen development against NeuGcGM3, a type of lipid, and a phase 2 and 3 study demonstrated significantly prolonged overall survival rates compared to the placebo (275). The TG4010, a viral vector vaccine, consists of a modified Vaccinia Virus Ankara (MVA) that carries the human MUC1 gene and interleukin 2 (276). In a phase I clinical trial, Rochlitz et al. observed a promising safety profile for TG4010, with one lung cancer patient experiencing significant reduction in metastasis over a 14-month period (277). A phase II randomized clinical trial suggests that combining TG4010 with chemotherapy in first-line advanced or metastatic NSCLC patients (stages IIIB and IV) may improve the efficacy of chemotherapy (278).

## 4 New strategies for prevention and combating immunotherapy resistance

### 4.1 Enhance T cell priming

New approaches to enhance T cell priming for T cell engineering and vaccination have emerged. These include Tumor-Infiltrating Lymphocyte (TIL) therapy, adoptive cell therapy utilizing Chimeric Antigen Receptor T cells (CAR-T cells), and personalized vaccines (279).

TIL therapy involves isolating TIL from tumor tissue, expanding and screening them to identify tumor antigens, and reintroducing them into the patient (279). Clinical trials are currently underway to compare the efficacy of TIL adoptive cell therapy alone or in combination with ICI in first-line and resistant NSCLC patients, with positive results demonstrating tumor regression and disease control in previously unresponsive patients (280). In contrast, CAR-T cells therapy involves genetically engineering T lymphocytes to express synthetic tumor antigen receptors, enabling them to recognize antigens independently of MHC presentation (255). Although adoptive cell therapy has shown promise in hematologic malignancies, its efficacy in solid tumors, including NSCLC, remains limited, highlighting the need for improved target antigens (255). Personalized vaccines based on tumor-specific neoantigens present another avenue for augmenting T cell-mediated anti-tumor responses. An ongoing study is evaluating the NEO-PV-01 personalized vaccine in combination with pembrolizumab and chemotherapy as a first-line (1L) treatment for NSCLC (NCT03380871) (). However, only a small fraction of mutations encode neoantigens recognizable by T cells. Other potential neoantigens include peptides resulting from single-nucleotide variations, insertions and deletions causing frameshifts, structural variations, and even post-translational events resulting from abnormal RNA splicing mediated by viral genome integration (281).

### 4.2 Overcoming the resistance to ICI

#### 4.2.1 Targeting VEGF

Due to the mechanical damage caused by VEGF-mediated angiogenesis and vessel remodeling, lymphocytes encounter impairments in their transendothelial migration process (282). VEGF, acting as an extracellular mediator, exerts significant influence on tumor immunity through diverse mechanisms. These include the inhibition of dendritic cell maturation and antigen presentation, upregulation of immune suppressive mediators such as PD-L1, PD-L2, IDO-1, IL-6, and IL-10, induction of regulatory T cells, and the accumulation of MDSCs (282). Several studies have investigated monoclonal antibodies and small molecules targeting the VEGF axis, aiming to restore VEGF-mediated MDSC infiltration and subsequent immune suppression (283). In the Impower 150 study, which focused on non-squamous NSCLC patients, the combination of anti-VEGF monoclonal antibody bevacizumab, atezolizumab, and chemotherapy as first-line treatment resulted in a significant prolongation of overall survival (OS) compared to chemotherapy alone (284). Additional drugs, including monoclonal antibodies such as ramucirumab and multitargeted kinase inhibitors like lenvatinib, sitravatinib, nintedanib, and axitinib, are currently undergoing evaluation for their efficacy in first-line and ICI-resistant NSCLC patients (244). In a phase Ib/II study involving 22 NSCLC patients (a majority of whom had received prior treatment, with 52% having received prior ICI therapy, the combination of lenvatinib and pembrolizumab showed promising preliminary results, with an objective response rate (ORR) of 33% and a median progression-free survival (PFS) of 5.9 months (285). Based on these encouraging findings, the LEAP series III clinical trials are currently underway to validate the effectiveness of lenvatinib and pembrolizumab as both first-line and later-line treatments (286). Moreover, a phase III study is investigating the efficacy of sitravatinib in combination with nivolumab for ICI-resistant patients (287).

#### 4.2.2 Cytokines

Research is underway to evaluate cytokines that can be specifically targeted. Notably, the use of the anti-IL-1β monoclonal antibody canakinumab has shown promising results in reducing NSCLC incidence and mortality in patients with atherosclerosis (288). This has sparked significant interest in IL-1β as a potential therapeutic target. However, the combination of canakinumab and docetaxel chemotherapy did not provide any benefits to ICI-resistant NSCLC patients, as observed in the phase III CANOPY-2 study (289). Currently, canakinumab is undergoing validation in phase III clinical trials as a first-line treatment in combination with immunotherapy and chemotherapy for adjuvant therapy, aiming to reduce immune resistance (290). On the other hand, studies targeting IL-10 and TGF-β have yielded disappointing results. In the phase II CYPRESS1 and CYPRESS2 studies, the use of pegilodecakin, a PEGylated recombinant human IL-10, in combination with ICI did not show benefits in terms of ORR and survival in NSCLC patients. Moreover, it led to significant adverse reactions (291). Similarly, despite the initial promising efficacy and safety of bintrafusp alfa, a bifunctional protein targeting TGF-β and PD-L1, it failed to provide benefits to ICI-naïve NSCLC patients with high PD-L1 expression compared to pembrolizumab in the phase III Lung-037 study. The trial was terminated recently due to futility (292). In addition to these findings, there is a growing body of research focusing on the gut microbiome as a potential target for immune anti-tumor therapy. Evidence suggests that the enrichment of different microbial communities and the use of broad-spectrum antibiotics can serve as predictors of ICI efficacy in NSCLC patients. This indicates that altering the composition of the gut microbiome, such as through fecal microbiota transplantation, could be a treatment strategy to enhance immunotherapy efficacy or reverse ICI resistance (293).

In summary, the degree and duration of immune anti-tumor response in cancer patients are dependent on the complexity of the immune system and its dynamic interaction with the TME, as well as the evolutionary process of tumor clone selection. However, resistance often emerges in patients at different stages, such as early in treatment, after a prolonged period of clinical benefit, or even after treatment discontinuation for any reason. These resistance mechanisms can take various forms. In addition, the factors influencing immune response and the underlying mechanisms of specific immune resistance are multifaceted. Currently, research focused on reducing resistance to ICI is still in its early stages. Most strategies primarily rely on biomarkers, which may limit the efficacy of newly developed therapies.

In fact, personalized therapies based on biomarkers and specific resistance patterns may offer the most promising approach for predicting, preventing, and mitigating ICI resistance. In addition to PD-L1, numerous other biomarkers have been evaluated for predicting ICI efficacy or resistance. These include molecular and genetic characteristics such as tumor mutation burden, neoantigen load, and selective gene mutations, as well as specific immune cell subtypes like TILs and MDSCs. Furthermore, immune signaling pathways have also been explored as potential biomarkers.

Advanced analytical tools can enable the development of highly complex multiparameter evaluation methods for the TME and biomarkers. By integrating genomic, transcriptomic, and spatial positioning data, these tools can facilitate the identification of patients who are more likely to benefit from immunotherapy. Here, we summarized the Phase III Clinical Studies of Anti-ICI Resistance Strategies In Advanced NSCLC in recent 5 years in **Table 2**.

**Table 2:** Phase III clinical studies of Anti-ICI resistance strategies in advanced NSCLC.

Study Name (NCT Number)	Research Scenarios	Target	Treatment Strategy	Study Endpoints
SKYSCRAPER-01 (NCT04294810)	1L, PD-L1 ≥ 50%	TIGIT	Atezolizumab plus Tiragolumab or Placebo	OS and PFS
MK-7684A-003 (NCT04738487)	1L, PD-L1 ≥ 1%	TIGIT	MK-7684A (vs. Pembrolizumab)	OS and PFS
INTR@PID-Lung-037 (NCT03631706)	1L, PD-L1 ≥ 50%	TGF-β and PD-L1	Bintrafusp alfa (vs. Pembrolizumab)	OS and PFS
LEAP-007 (NCT03829332)	1L, PD-L1 ≥ 1%	VEGF (RTK)	Pembrolizumab plus Lenvatinib or Placebo	OS and PFS
LEAP-006 (NCT03829319)	1L (N-sq)	VEGF (RTK)	Platinum and Pemetrexed and Pembrolizumab and Lenvatinib or Placebo	DLT, OS and PFS
CANOPY-1 (NCT03631199)	1L	IL-1β	Platinum and Pemetrexed and Pembrolizumab and Canakinumab or Placebo	OS and PFS
KEYLYNK-006 (NCT03976323)	1L, Maintenance (N-sq)	PARP	Platinum and Pemetrexed and Pembrolizumab → Pembrolizumab and Olaparib (vs. Pemetrexed)	OS and PFS
KEYLYNK-008 (NCT03976362)	1L, Maintenance (Sq)	PARP	Platinum and Abraxane and Pembrolizumab → Pembrolizumab and Olaparib (vs. Pemetrexed)	OS and PFS
LEAP-008 (NCT03976375)	Post-Chemotherapy and ICI	VEGF (RTK)	Pembrolizumab plus Lenvatinib (vs. Docetaxel)	OS and PFS
SAPPHIRE (NCT03906071)	Post-Chemotherapy and ICI	VEGF (RTK)	Nivolumab and Sitravatinib (vs. Docetaxel)	OS
CONTACT-01 (NCT04471428)	Post-Chemotherapy and ICI	c-MET (RTK)	Atezolizumab and Cabozantinib (vs. Docetaxel)	OS
CANOPY-2 (NCT03626545)	Post-Chemotherapy and ICI	IL-1β	Docetaxel and Canakinumab or Placebo	OS

### 4.3 Overcoming the resistance to CTLA-4 immunotherapy

CTLA-4, also known as CD152, is a transmembrane protein expressed in activated CD4+ and CD8+ T cells (294–298). Under physiological conditions, CTLA-4 and CD80/CD86 binding can inhibit T-cell activation signals and prevent autoimmune disease (299, 300). Blocking CTLA-4 can directly target inhibitory signals on effector T cells and reduce the inhibitory effect of regulatory T cells (Tregs) (301–305) thus effectively enhancing the antitumor effect of T cells.

However, while immune checkpoint blocking therapies (ICBs) are revolutionizing therapeutic algorithms for cancers, the frequently observed innate, adaptive or acquired drug resistance remains an inevitable obstacle to a durable antitumor activity, thus leading to non-response or tumor relapse.

Increased Tregs and dendritic cells (DCs) in the tumor environment may be responsible for acquired resistance and provide another therapeutic target to prevent or overcome resistance. Tregs can be identified with the cell surface markers forkhead box protein 3 (Foxp3), CD25, CD357, lymphocyte-activation gene 3 (LAG3), CTLA-4, and low CD127. Foxp3 are crucial in the immunosuppressive activity of suppressor T cells or Tregs within the lung cancer TME. Foxp3 is a transcription factor that is upregulated in TILs and tumor cells and coveys a negative prognostic factor in the NSCLC and maybe a future target for resistant tumors. LAG3 is also a co-inhibitory molecule on TILs, Tregs, DCs, and NK cells that dampens T cell activation via its binding to MHC II receptors, making it another possible therapeutic target after resistance. T cell immunoglobulin and mucin domain-3-containing molecule 3 (TIM3) is a cell surface protein typically seen on DCs that interacts with Galectin-9 on T cells leading to inhibition of the T cell response. TIM-3 expression can be seen on TILs, and its interaction with galectin-9 on Tregs or tumor cells can lead to T cell inhibition (306). Increased TIM-3 expression has been seen as a marker of poor prognosis but may also provide an alternative checkpoint target for therapy after PD-1 failure (306, 307).

In order to overcome resistance, potential therapeutic strategies include enhancing antigen procession and presentation, reinforcing the activity and infiltration of T cells, and destroying immunosuppression microenvironment. In future, determining the driving factors behind ICB resistance by tools of precision medicine may maximize clinical benefits from ICBs. Moreover, efforts in individualized dosing, intermittent administration and/or combinatory regimens have opened new directions for overcoming ICB resistance.

Above all, in recent 5 years, the promising strategies for overcoming the resistance to CTLA-4 immunotherapy in NSCLC can be listed in **Table 3**.

**Table 3:** The promising strategies for overcoming the resistance to CTLA-4 immunotherapy of NSCLC in recent 5 years.

Resistant to	Resistance Mechanisms	Target	Promising Strategies	Reference
Ipilimumab	tumor mutations and adaptations	Interferon-gamma (IFNγ) pathway	combination with other inhibitors	(308)
Ipilimumab	tumor mutations and adaptations	VEGF	Ipilimumab + Bevacizumab	(309)
Ipilimumab	tumor mutations and adaptations	VEGFR TKI	combination with VEGFR TKI	(285)
Ipilimumab	Regulating MDSCs	CK2	Ipilimumab + ATRA	(310)
Ipilimumab	hypoxia, epithelial to mesenchymal transition (EMT), and extracellular matrix remodeling	TGF-β	combination with other inhibitors	(311)
Ipilimumab	Reducing MAPK-dependent chemokine production	–	combination with other Sema4D mAb	(312)
Ipilimumab	amplify antitumor T-cell responses	PD-L1	Ipilimumab + pembrolizumab	(313)
Tremelimumab	amplify antitumor T-cell responses	PD-L1	Tremelimumab + Durvalumab	(314)
Tremelimumab	KRAS-mutant NSCLC	MEK	Tremelimumab + selumetinib+ durvalumab	(315)

### 4.4 Overcoming the resistance to cancer vaccines

Despite the potential benefits observed in NSCLC treatment, resistance to cancer vaccines remains a significant challenge. Resistance to cancer vaccines can arise through tumor intrinsic and extrinsic resistance. Tumor intrinsic resistance encompasses six aspects: mutations in signaling pathways supporting tumor-immune control, loss of tumor antigen expression, changes in antigen processing pathways, loss of HLA expression, epigenetic changes, and increased expression of immunosuppressive ligands (316–318). Extrinsic resistance to cancer vaccines may be caused by immunosuppressive cells (such as CAFs, MDSCs, Tregs, and M2 macrophages) and immunosuppressive cytokines, which can directly or indirectly inhibit the activation of effector T cells and DC-mediated T cells in TME (319–323). Lung cancer cells have been shown to produce a variety of immunosuppressive molecules, including TGF-β, prostaglandin E2, IL-10, and cyclooxygenase-2, which can affect DC processing and presentation, as well as the acquisition and expression of CTL effector cell function (324–326). Strategies for developing effective immunotherapy for NSCLC have previously faced challenges. However, recent successes in identifying T cell responses to tumor-specific antigens in patients with NSCLC suggest that this goal may now be achievable (327). The identification of lung tumor-associated antigens and presenting them in the optimal context may enable the immune system to generate anti-lung tumor effector cells (328).

Researchers are currently exploring various strategies to overcome resistance. Some representative strategies include:

1) Combination therapies: Clinical trials have been initiated to explore the combination of ICBs with various established cancer vaccines in the hope of providing more effective tumor-specific T-cell responses in patients with advanced-stage NSCLC. These include the combination of TG4010, nivolumab and anti-PD1 antibody (329); the CIMAvax-EGF vaccine with anti-PD1 antibodies (273); and the melanoma antigen family A, 3 (MAGE-A3) vaccine with ICBs (330).
2) Personalized vaccines: The vaccine Vx-001, targeting TERT, a tumor antigen derived from universal tumor antigens and recognized as neoantigens, showed strong immunogenicity. However, a phase II clinical trial in NSCLC patients did not demonstrate any significant improvements in overall survival, disease control rate, or time to treatment failure (331). Linette et al. present the development of a clinical grade neoantigen vaccine formulation (FRAME-001) with stability for up to 32 weeks, intended for immunotherapy in advanced NSCLC in combination with pembrolizumab, showcasing the potential of personalized therapeutic cancer vaccines targeting tumor-specific neoantigens (332).
3) Modulating the TME: Cancer vaccines that target tumor-associated antigens (TAAs) may face challenges due to cancer cell evasion mechanisms, making the stable components of the cancer microenvironment an alternative vaccination target to avoid immune anergy caused by genetic mutations (333). The utilization of combined cancer vaccines alongside diverse immunotherapies or standardized therapies has emerged as an effective approach in recent years to counteract the immunosuppressive TME and enhance clinical outcomes (334). The field of NSCLC treatment is currently divided into three main categories, which are DC vaccines (NCT05195619, NCT03546361), DNA-based vaccine(NCT05242965), peptide-based cancer vaccines (NCT05254184, NCT03879694, NCT03633110).
4) Adjuvants and immune stimulants: The combination of a DNA vaccine containing a fusion gene of MUC1 and VEGFR2, along with the use of GM-CSF as an adjuvant showed an increased inhibition in the growth of MUC1-expressing tumours and prolonged mouse survival (335). A combination vaccine involving irradiated lung adenocarcinoma cells and transfected K562 cells with hCD40L and hGM-CSF induced tumor regression in metastatic lung adenocarcinoma by activating dendritic cells, resulting in a median overall survival of 7.9 months and median progression-free survival of 1.7 months, with 5 out of 14 patients showing CD8+ T-cell activation after vaccination (336). In a phase I study, intratumoral administration of autologous DC transduced with an adenoviral vector expressing the CCL21 gene (Ad-CCL21-DC) in patients with advanced NSCLC resulted in increased tumor CD8+ T-cell infiltration, induction of systemic tumor antigen-specific immune responses, and enhanced tumor PD-L1 expression (337).
5) mRNA-based vaccines: CV9201 and CV9202 have been investigated in NSCLC (338). In particular, CV9202 encoding six tumor-associated antigens(NY-ESO-1, MAGE-C1, MAGE-C2, Survivin, 5T4 and MUC) demonstrated good tolerance and elicited antigen-specific immune responses in a phase Ib study of stage IV NSCLC patients (339).

Despite these limitations, lung cancer vaccines continue to be a promising and actively investigated area. Recent phase III trials targeting specific subpopulations with L-BLP25, MAGE-A3, and belagenpumatucel-L vaccines have demonstrated potential benefits, highlighting the importance of combining vaccines with chemotherapy and radiation as part of a multimodal approach to target lung cancer. Consulting with a healthcare professional or an oncologist can provide the most up-to-date information regarding the treatment options available for lung cancer and drug resistance. Here, we summarized the promising strategies to overcoming the resistance of cancer vaccines to NSCLC in the past 5 years in **Table 4**.

**Table 4:** The promising strategies to overcome drug resistance with cancer vaccines to NSCLC.

Mechanisms to improve resistance	Target population	Target	Promising Strategies for resistance	Clinical Trial number	Reference
Reduce circulating EGF levels and decrease its binding to EGFR on cancer cells	Stage IIIB/IV NSCLC patients following front-line CTP	EGF	CIMAvax-EGF vaccine	RPCEC00000161	(273, 340)
Augment tumor-specific CD8+ T cell proportions and, activate their cytotoxic action	NSCLC patients without response to ICIs	MUC1	Combination of TG4010 and nivolumab	NCT02823990	(329)
Induce antigen-specific T cell immune response and increase anti-tumor activity of CD8+ T cells	Stage III/IV NSCLC patients	Patient-specific neoantigen peptides	FRAME-001	NCT04998474	(332)
By activating DC cells (via cytokine GM-CSF) and enhancing CD40L expression, the vaccine aims to increase DC activity in local vaccine sites.	Patients with refractory lung adenocarcinoma	CD40L, GM-CSF	DNA vaccine	NCT00601796	(336)
Recruit lymphocytes and antigen-stimulated DCs, leading to increased infiltrates of CD4, CD8, and CD11c+DEC205+ dendritic cells in the tumor, creating a lymphoid-like microenvironment and facilitating cognate T cell activation.	Stage IIIB/IV NSCLC patients	CCL21	DC vaccine	NCT01574222	(337)
Inducing antigen-specific cellular and/or humoral immune responses via targeting specific tumor-associated antigens (TAAs)	Stage IV NSCLC patients	6 non-small cell lung cancer (NSCLC)-associated antigens (NY-ESO-1, MAGE-C1, MAGE-C2, survivin, 5T4, and MUC-1)	mRNA vaccine (CV9202)	NCT01915524	(338)
	Patients with stage IIIB/IV NSCLC who have achieved at least stable disease following first-line chemotherapy or chemoradiation	5 non-small cell lung cancer-antigens: New York esophageal squamous cell carcinoma-1, melanoma antigen family C1/C2, survivin, and trophoblast glycoprotein	mRNA vaccine (CV9201)	NCT00923312	(339)
Stimulates T cell priming and infiltration, triggers local immune responses, and counteracts cancer-induced immune evasion within the tumor microenvironment.	Stage I/II NSCLC patients	MAGE-A3	MAGE-A3 vaccine with ICBs	NCT02879760	(341)
Stimulates the immune system to generate antibodies that specifically produce antibodies against hTERT and attack against hTERT-expressing cancer cells.	Patients with resistance to immune checkpoint inhibitors (ICIs) and non-immunogenic cold NSCLCs	TERT (TElomerase Reverse Transcriptase)	Vx-001	NCT01935154	(342, 343)

### 4.5 New strategy: immune system-modulators

Immune system modulators play a crucial role in cancer treatment, encompassing drugs and proteins. These components function by either augmenting the immune system’s capacity to identify and combat cancer cells or inhibiting signals that enable cancer cells to elude immune detection. Examples of such modulators include cytokines, BCG, and various other drugs (344).

In the context of lung cancer, immunomodulators have been employed to enhance the immune response against cancer cells, potentially leading to improved patient outcomes. Apart from checkpoint inhibitors, another class of immunomodulator utilized in lung cancer treatment is cytokines. Numerous cytokine therapies have been investigated for their efficacy in treating NSCLC. Among these, interleukin-2 (IL-2) is one of the most extensively studied cytokines. IL-2 has been demonstrated to stimulate the immune system, specifically the activation and proliferation of T cells. Its therapeutic application has been explored in both NSCLC and SCLC (345–348). For instance, IL-2 therapy, such as ALKS 4230, has shown promise in improving survival rates among patients with advanced NSCLC (349). In the case of NSCLC, IL-2 has been administered in combination with other treatments, such as chemotherapy and radiation therapy, yielding mixed results (350). Some studies have reported enhanced response rates and overall survival, while others have not observed significant benefits (351). Additionally, patients with IL-2-expressing tumors have exhibited significantly improved 5-year overall survival rates in cases of radically resected NSCLC (352). Other cytokines, including IFN-α and IFN-γ, have also been investigated for their potential in NSCLC treatment. These cytokines have demonstrated antitumor effects and the ability to stimulate the immune system, yet their application in lung cancer remains investigational (345, 353, 354). Furthermore, Bacille Calmette-Guérin (BCG), a live attenuated vaccine traditionally used for tuberculosis prevention, has found application in cancer treatment. BCG can be injected directly into the lungs to stimulate an immune response against cancer cells. It can be administered alone or in conjunction with other therapies, such as chemotherapy or radiation therapy (355).

Currently, the FDA has approved 16 different immunomodulators for the treatment of various cancer types. These immunomodulators include nine checkpoint inhibitors, four cytokines, two adjuvants, and a small molecule with immunomodulatory properties. Most of these approvals are for advanced or resistant cancers, but recently some have also been approved as first-line systemic treatments for certain metastatic cancers. Among the approved cytokine drugs are Aldesleukin (Proleukin^®^), Granulocyte-macrophage colony-stimulating factor (GM-CSF), Interferon alfa-2a, Interferon alfa-2b (Intron A^®^), and Peginterferon alfa-2b (Sylatron^®^/PEG-Intron^®^) (356). In addition, adjunct agents such as Imiquimod and Poly ICLC (Hiltonol^®^) have been used in cancer treatment (357, 358).

## 5 Conclusion

In this review, we discuss the mechanisms of resistance to targeted therapy and immunotherapy in NSCLC and outline promising strategies for overcoming resistance from different perspectives.

For targeted therapy, we categorized numerous NSCLC-related targets into common and uncommon clinical gene targets. EGFR is the predominant oncogene in NSCLC, with resistance mechanisms involving EGFR-dependent and EGFR-independent pathways. Clinical evidence supports that resistance to the classical C797S/T790M mutation can be overcome by combining first- and third-generation EGFR TKIs. Various mutation types can be targeted using a combination of EGFR-TKIs and chemotherapy, or specific small molecule inhibitors. Furthermore, fourth-generation EGFR-TKIs and ADCs have been identified as successful treatment approaches to address resistance. Resistance to KRAS inhibitors develops due to the adaptive reactivation of the RAS pathway, the presence of non-G12C KRAS mutations, and the activation of alternative pathways. Employing combination therapy is the primary method to address resistance. Several ALK-TKIs have been authorized for treating global ALK-positive patients with the ALK gene, but they may lead to ALK-TKI resistance by activating the bypass signaling pathway. Combining an ALK-TKI with an immune checkpoint inhibitor can help overcome resistance. ROS1 resistance may develop from intrinsic factors like gene fusions or mutations leading to continuous activation of ROS1 kinase, and extrinsic factors such as activation of alternative signaling pathways. Clinical trials are assessing new ROS1 inhibitors for ALK and ROS1 fusion-positive NSCLC cancers. Research is also exploring the combination of ROS1-targeted drugs with other treatments to enhance effectiveness. BRAF is also a commonly targeted gene, with the V600E mutation being the most prevalent type in NSCLC. However, the mechanisms of resistance to BRAF inhibitors in NSCLC are not completely understood. The more uncommon gene targets include RET, NRAS, PIK3CA, HER2, and c-MET. PIK3CA is commonly mutated and amplified in various cancer types. There are no approved targeted treatments for PIK3CA. Research on PIK3CA resistance has mainly concentrated on breast cancer cells, with limited focus on understanding resistance mechanisms in NSCLC. The FDA has approved T-DXd, an ADC, for treating patients with HER2-mutated NSCLC. The first c-MET single-target inhibitor in the world was authorized for treating patients with advanced NSCLC harboring a MET exon 14 skipping mutation.

Immunotherapy employing ICIs that target the PD-L1/PD-1 axis and CTLA-4 has shown significant improvements in the survival of lung cancer patients. However, drug resistance remains a significant issue, with the TME playing a crucial role. Various strategies are employed to combat resistance to ICI therapy. For instance, enhancing T cell priming through strategies including TIL therapy, CAR-T cell therapy, and personalized vaccines, targeting VEGF, or using cytokines. ICBs are revolutionizing cancer treatment. Potential strategies to overcome resistance involve improving antigen processing and delivery, enhancing T-cell activity and infiltration, and disrupting the immunosuppressive microenvironment. Cancer vaccines are a significant treatment method for NSCLC. They can target tumor antigens, minimize side effects, and can be combined with other treatments like immune checkpoint inhibitors or targeted therapies. Such vaccinations can be used at all stages of NSCLC, either as an additional therapy after surgery or as a treatment for metastatic disease. Immune system modulators have been utilized to enhance the immune response against lung cancer cells. The FDA has approved 16 immunomodulators for treating different types of cancer, with IL-2 being an extensively researched cytokine.

In conclusion, while many patients with NSCLC still benefit from target therapy and immunotherapy, the resistance to targeted therapy and immunotherapy is a significant challenge and still poses challenges in the treatment of NSCLC. However, combination therapies that target multiple pathways or mechanisms and biomarker identification-based approaches show promise for overcoming resistance. Comprehending the underlying mechanisms of resistance and the development of novel therapies are essential for devising successful approaches. Ongoing research in these fields will help overcome resistance and hold promise for improving patient outcomes in the future.

## Author contributions

YX: Writing – original draft, Writing – review & editing. XL: Writing – original draft, Writing – review & editing. YW: Writing – original draft, Writing – review & editing. DZ: Writing – original draft. QM: Writing – original draft. LJ: Writing – original draft. SY: Writing – original draft. SZ: Writing – original draft. XZ: Writing – original draft. YL: Writing – original draft, Writing – review & editing. BW: Writing – original draft, Writing – review & editing.

## Glossary

**Table:**

NSCLC	non-small cell lung cancer
EGFR	epidermal growth factor receptor
ALK	anaplastic lymphoma kinase
ROS1	proto-oncogene tyrosine-protein kinase-1
MET	mesenchymal to epithelial transition factor
KRAS	kirsten rat sarcoma viral oncogene
TKIs	tyrosine kinase inhibitors
MAPK	mitogen-activated protein kinase
PI3K	phosphatidylinositol 3-kinase
HER2	human epidermal growth factor receptor 2
FGFR	fibroblast growth factor receptor
BRAF	B-Raf proto-oncogene
RET	ret proto-oncogene
NTRK	neurotrophic tropomyosin kinase receptors
PIK3CA	phosphatidylinositol 4,5-bisphosphate 3-kinase catalytic subunit alpha
SFK	Src family kinase
FAK	focal adhesion kinase
SCLC	small cell lung carcinoma
EMT	epithelial-to-mesenchymal transition
SCCT	squamous cell carcinoma transition
ATP	Adenosine triphosphate
PFS	progression-free survival
MEK	mitogen-activated protein kinase kinas
RTKs	receptor tyrosine kinases
YAP 1	Yes-associated protein 1
ABCC11	ATP-binding cassette subfamily C member 11
NF2	neurofibromatosis type 2
NRAS	neuroblastoma rat sarcoma virus oncogene homolog
PTEN	phosphatase and tensin homolog
CTCs	circulating tumor cells
ICI	immune checkpoint inhibitors
TME	tumor microenvironment
MDSCs	myeloid-derived suppressor cells
TIL	Tumor-Infiltrating Lymphocyte
CAR-T cells	Chimeric Antigen Receptor T cells
PD-L1	programmed death-ligand 1
CTLA-4	cytotoxic T-lymphocyte-associated antigen 4
Tregs	regulatory T cells
DCs	dendritic cells
Foxp3	Forkhead box protein 3
LAG3	lymphocyte-activation gene 3
TIM3	mucin domain-3-containing molecule 3
TAAs	tumor-associated antigens
1L	first-line
DLT	dose-limiting toxicity
IL	interleukin
MK-7684A	pablizumab/vibolizumab combination
N-Sq	non-squamous cancer
OS	overall survival
PARP	polyadenosine diphosphate ribose polymerase
PFS	progression-free survival
RTK	receptor tyrosine kinase
Sq	squamous carcinoma
TGF-β	transforming growth factor-β
TIGIT	T-cell immunoglobulin and ITIM structural domain
VEGF	Vascular Endothelial Growth Factor
MAGE-A3	melanoma antigen family A, 3
CTA	cancer/testis antigen
LUSC	squamous cell carcinoma
DFS	disease-free survival
MVA	Vaccinia Virus Ankara
